# Supplementary material for: From amino acid mixtures to peptides in liquid sulphur dioxide on early Earth
Source: Nat Commun. 2021 Dec 10;12:7182. doi: 10.1038/s41467-021-27527-7 (PMC8664857; doi:10.1038/s41467-021-27527-7)
Supplement: Supplementary file 1 — Supplementary Information [file 41467_2021_27527_MOESM1_ESM.docx]

Supplementary Information

From amino acid mixtures to peptides
in liquid sulphur dioxide on early Earth

*Fabian Sauer, Maren Haas, Constanze Sydow, Alexander F. Siegle, Christoph A. Lauer and Oliver Trapp*

**Contents**

[1 Methods 3](#_Toc69480472)

[1.1 Materials 3](#_Toc69480473)

[1.2 Composition of amino acid mixtures 3](#_Toc69480474)

[1.3 Pressure apparatus 4](#_Toc69480475)

[1.4 Preparation of linear polyacrylamide-coated capillaries 4](#_Toc69480476)

[1.5 Removal of copper ions 5](#_Toc69480477)

[1.6 High pressure liquid chromatography-tandem mass spectrometry analysis 5](#_Toc69480478)

[1.7 Elemental analysis of minerals 6](#_Toc69480479)

[2 Quantitative investigations 7](#_Toc69480480)

[2.1 Calibration plots 7](#_Toc69480481)

[2.2 Peptide formation in SO_2_ 10](#_Toc69480482)

[2.2.1 400 mM, 21 d 10](#_Toc69480483)

[2.2.2 400 mM, 7 d 12](#_Toc69480484)

[2.2.3 400 mM, 3 d 14](#_Toc69480485)

[2.2.4 400 mM, 1 d 16](#_Toc69480486)

[2.2.5 50 mM, 7 d 18](#_Toc69480487)

[2.2.6 100 mM, 7 d 20](#_Toc69480488)

[2.2.7 200 mM, 7 d 22](#_Toc69480489)

[3 Dipeptide product spectra 24](#_Toc69480490)

[4 Extracted ion electropherograms and mass spectra 37](#_Toc69480491)

[5 Extracted ion chromatograms and mass spectra 74](#_Toc69480492)

[6 References 76](#_Toc69480493)

# Methods

## Materials

All chemicals were purchased from commercial suppliers (Sigma-Aldrich, TCI, Alfa Aesar) in analytical grade and used without further purification. Water was deionized by a VWR Puranity PU 15 (VWR, Leuven, Belgium). Sulphur dioxide (SO_2_) N38 (99.98 %) was purchased from Air Liquide Germany.

## Composition of amino acid mixtures

**Full mixture:** l-alanine (115.8 mg, 1.3 mmol, 1.0 eq.), l-arginine (226.5 mg, 1.3 mmol, 1.0 eq.), l-asparagine (171.8 mg, 1.3 mmol, 1.0 eq.), l-aspartic acid (173.0 mg, 1.3 mmol, 1.0 eq.), l-cysteine (157.5 mg, 1.3 mmol, 1.0 eq.), l-glutamine (190.0 mg, 1.3 mmol, 1.0 eq.), l-glutamic acid (191.3 mg, 1.3 mmol, 1.0 eq.), glycine (97.6 mg, 1.3 mmol, 1.0 eq.), l-histidine (201.7 mg, 1.3 mmol, 1.0 eq.), l-isoleucine (170.5 mg, 1.3 mmol, 1.0 eq.), l-leucine (170.5 mg, 1.3 mmol, 1.0 eq.), l-lysine (190.0 mg, 1.3 mmol, 1.0 eq.), l-methionine (194.0 mg, 1.3 mmol, 1.0 eq.), l-phenylalanine (214.7 mg, 1.3 mmol, 1.0 eq.), l-proline (149.7 mg, 1.3 mmol, 1.0 eq.), l-serine (136.6 mg, 1.3 mmol, 1.0 eq.), l-threonine (154.9 mg, 1.3 mmol, 1.0 eq.), l-tryptophan (266.8 mg, 1.3 mmol, 1.0 eq.), l-tyrosine (235.5 mg, 1.3 mmol, 1.0 eq.) and l-valine (152.3 mg, 1.3 mmol, 1.0 eq.).

**Non-polar mixture:** l-alanine (267.3 mg, 3.0 mmol, 1.0 eq.), l-isoleucine (393.5 mg, 3.0 mmol, 1.0 eq.), l-leucine (393.5 mg, 3.0 mmol, 1.0 eq.), l-methionine (447.6 mg, 3.0 mmol, 1.0 eq.), l-phenylalanine (495.6 mg, 3.0 mmol, 1.0 eq.), l-proline (345.4 mg, 3.0 mmol, 1.0 eq.), l-tryptophan (615.7 mg, 3.0 mmol, 1.0 eq.) und l-valine (351.4 mg, 3.0 mmol, 1.0 eq.).

**Polar, neutral mixture:** l-asparagine (528.5 mg, 4.0 mmol, 1.0 eq.), l-cysteine (484.6 mg, 4.0 mmol, 1.0 eq.), l-glutamine (584.6 mg, 4.0 mmol, 1.0 eq.), glycine (300.3 mg, 4.0 mmol, 1.0 eq.), l-serine (420.4 mg, 4.0 mmol, 1.0 eq.), l-threonine (476.5 mg, 4.0 mmol, 1.0 eq.) and l-tyrosine (724.8 mg, 4.0 mmol, 1.0 eq.).

**Alkaline mixture:** l-arginine (1306.5 mg, 7.5 mmol, 1.0 eq.), l-histidine (1163.7 mg, 7.5 mmol, 1.0 eq.) and l-lysine (1096.4 mg, 7.5 mmol, 1.0 eq.).

**Acidic mixture:** l-aspartic acid (1663.8 mg, 12.5 mmol, 1.0 eq.) and l-glutamic acid (1839.1 mg, 12.5 mmol, 1.0 eq.).

**Prebiotic mixture:** l-alanine (267.3 mg, 3.0 mmol, 1.0 eq.), l-aspartic acid (399.3 mg, 3.0 mmol, 1.0 eq.) und l-glutamic acid (441.4 mg, 3.0 mmol, 1.0 eq.) glycine (225.2 mg, 3.0 mmol, 1.0 eq.), l-isoleucine (393.5 mg, 3.0 mmol, 1.0 eq.), l-leucine (393.5 mg, 3.0 mmol, 1.0 eq.), l-proline (345.4 mg, 3.0 mmol, 1.0 eq.), l-serine (315.3 mg, 3.0 mmol, 1.0 eq.), l-threonine (357.4 mg, 3.0 mmol, 1.0 eq.) and l-valine (351.4 mg, 3.0 mmol, 1.0 eq.).

## Pressure apparatus


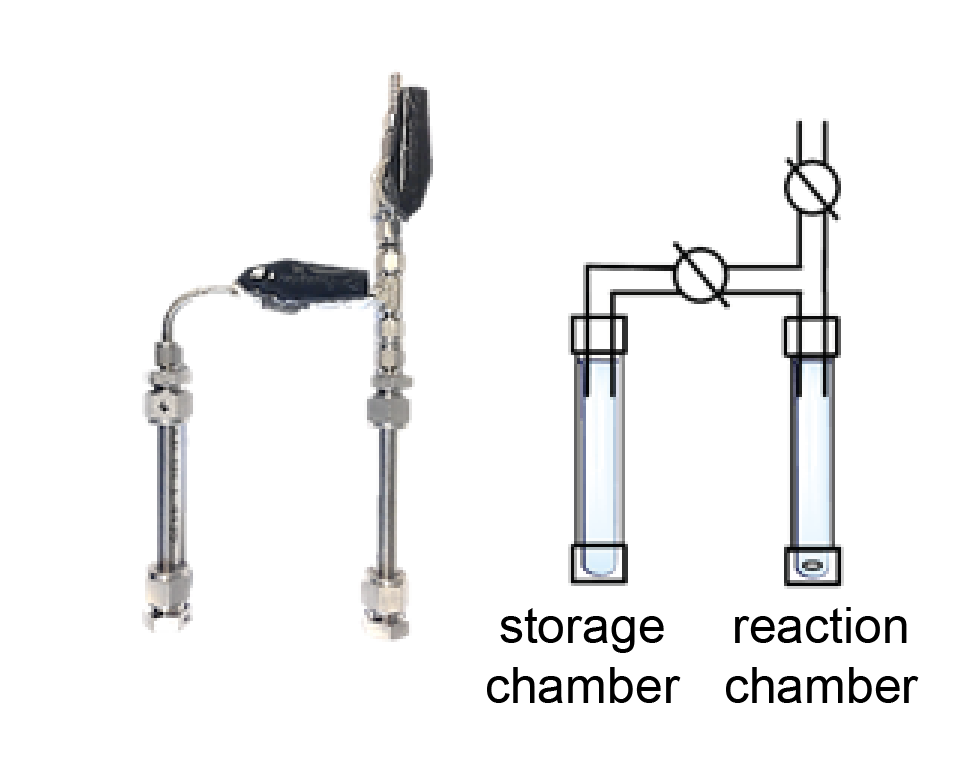


Supplementary Figure 1: Stainless steel pressure apparatus for reactions in SO_2_: storage unit and reaction chamber equipped with 3.5 mL glass test tube and magnetic stir bar.

## Preparation of linear polyacrylamide-coated capillaries

The linear polyacrylamide (LPA) coating was applied using a method similar to the one described by Dovichi et al.^[[1]](#endnote-1)^ A bare fused silica capillary (inner diameter = 0.05 mm, MicroQuartz, München) was therefor heated in a Thermo Focus GC oven at 200 °C and 30 kPa nitrogen pressure for 10 h. Subsequently, it was flushed with the following solvents by applying 5 bar nitrogen pressure: 0.1 M NaOH_(aq)_, 2 h; H_2_O, 30 min; 0.1 M HCl_(aq)_, 24 h; H_2_O, 1 h; N_2_ drying, 6 h. The capillary was filled with a solution of 200 μL γ-methacryloxypropyltrimethoxysilane (γ-MAPS) in 200 μL methanol, the ends sealed and the capillary heated at 45 °C for 6 h. After several rinsing steps (MeOH, 1 h; H_2_O, 1 h; N_2_ drying, 2 h) a polymerisation mixture consisting of 120 mg acrylamide in 3 mL H_2_O with addition of 10 μL of a solution containing 20 mg ammonium persulfate (APS) in 4 mL H_2_O was prepared. The mixture was vortexed and degassed with nitrogen for 10 min. The capillary was filled with the polymerization mixture, the ends sealed and heated for 30 min at 50 °C. After rinsing for 2 h with H_2_O, the capillaries were dried with nitrogen flow for another 2 h and stored until measurements.

## Removal of copper ions

Prior to capillary electrophoresis-tandem mass spectrometry analysis (CE-MS/MS) copper ions were removed with the ion exchange resin Amberlite IRC-748 (particle size 0.5-0.65 mm, Na^+^-form). The resin was washed with H_2_O, transferred to its H^+^-form with HCl (2 M) and washed with H_2_O until a neutral pH was reached. 5 mg of the reaction mixture was dissolved in 10 mL H_2_O and 30 mg of the ion exchange resin was added. After overnight stirring, the ion exchange resin was exchanged with 30 mg fresh resin and again stirred overnight. The resin was removed and the sample diluted with H_2_O for analysis.

## High pressure liquid chromatography-tandem mass spectrometry analysis

The reaction analysis by CE-MS/MS was verified for the system containing only glycine and l-alanine by high pressure liquid chromatography-tandem mass spectrometry (HPLC-MS/MS). An Agilent Technologies 1260 Infinity II SFC-uHPLC-Hybrid-System coupled to the 6550 iFunnel-Q-TOF mass spectrometer with an ESI interface was used. The separation was performed on a Daicel DCpak PTZ column (4.6x150 mm, 3 μm) at T = 30 °C using a flow of 0.9 mL/min. Eluent A: 20 mM NH_4_OAc_(aq)_ and B: acetonitrile were used with the gradient 70 % B (0-10 min), 70-0 % B (10-25 min), 0 % B (25-35 min). The injection volume was 10 μL. Mass spectra were recorded in the range of *m/z* 50 – 1500, using 120 V fragmentor voltage and collision induced dissociation at 15 eV. For data evaluation Agilent MassHunter Qualitative Analysis 10.0 software was used.

## Elemental analysis of minerals

Covellite (CuS, Poland) was purchased from Seltene Mineralien Gunnar Färber (Samswegen, Germany). Its composition was confirmed by ICP-OES measurements, which were performed by Jaroslava Obel (analytical services, chemistry department, LMU Munich) on a Varian Vista RL: Al 3.8 mg/g, Ba 0.1 mg/g, Ca 12.7 mg/g, Co 0.2 mg/g, Cu 585.7 mg/g, Fe 2.3 mg/g, Mg 6.9 mg/g, Mn 0.3 mg/g, Na 1.2 mg/g, S 284.9 mg/g, Si 4.9 mg/g.

# Quantitative evaluation of dipeptide formation in SO_2_

## Calibration plots


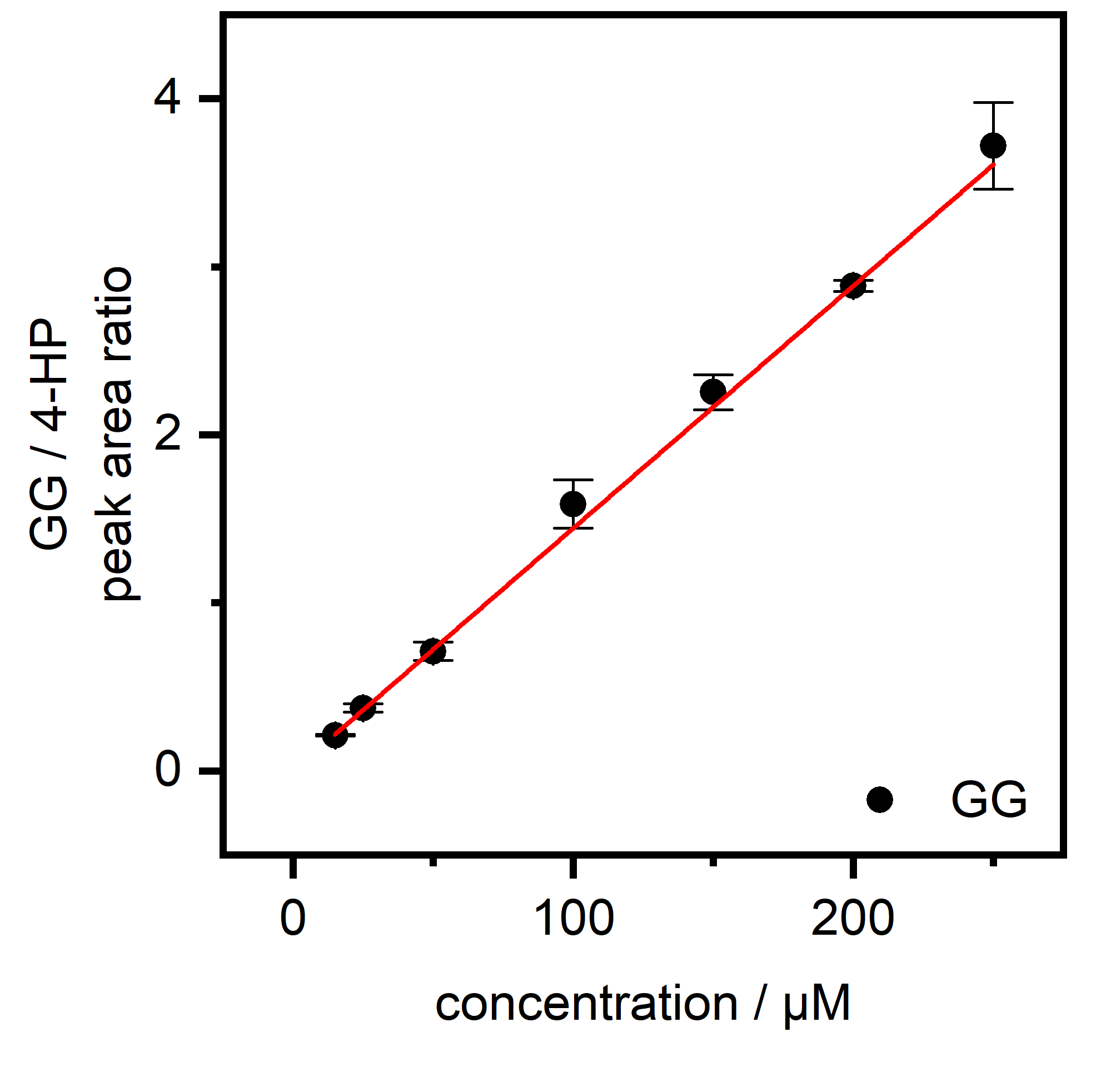


Supplementary Figure 2: Calibration plot of diglycine (GG) (m = 0.01443 ± 9.89762E-5, R^2^ = 0.99972) after the electrophoretic separation of a standard mixture consisting of diglycine, alanylglycine, glycylalanine and dialanine. The calibration curve was recorded in triplicates using a conductivity detector and 4-hydroxyproline (4-HP) as internal standard (100 µM). Bare fused silica capillary, 80 cm; BGE, AcOH (2 M); CE inlet, 30 kV; T = 25 °C, sample injection using 30 mbar pressure for 10 s.


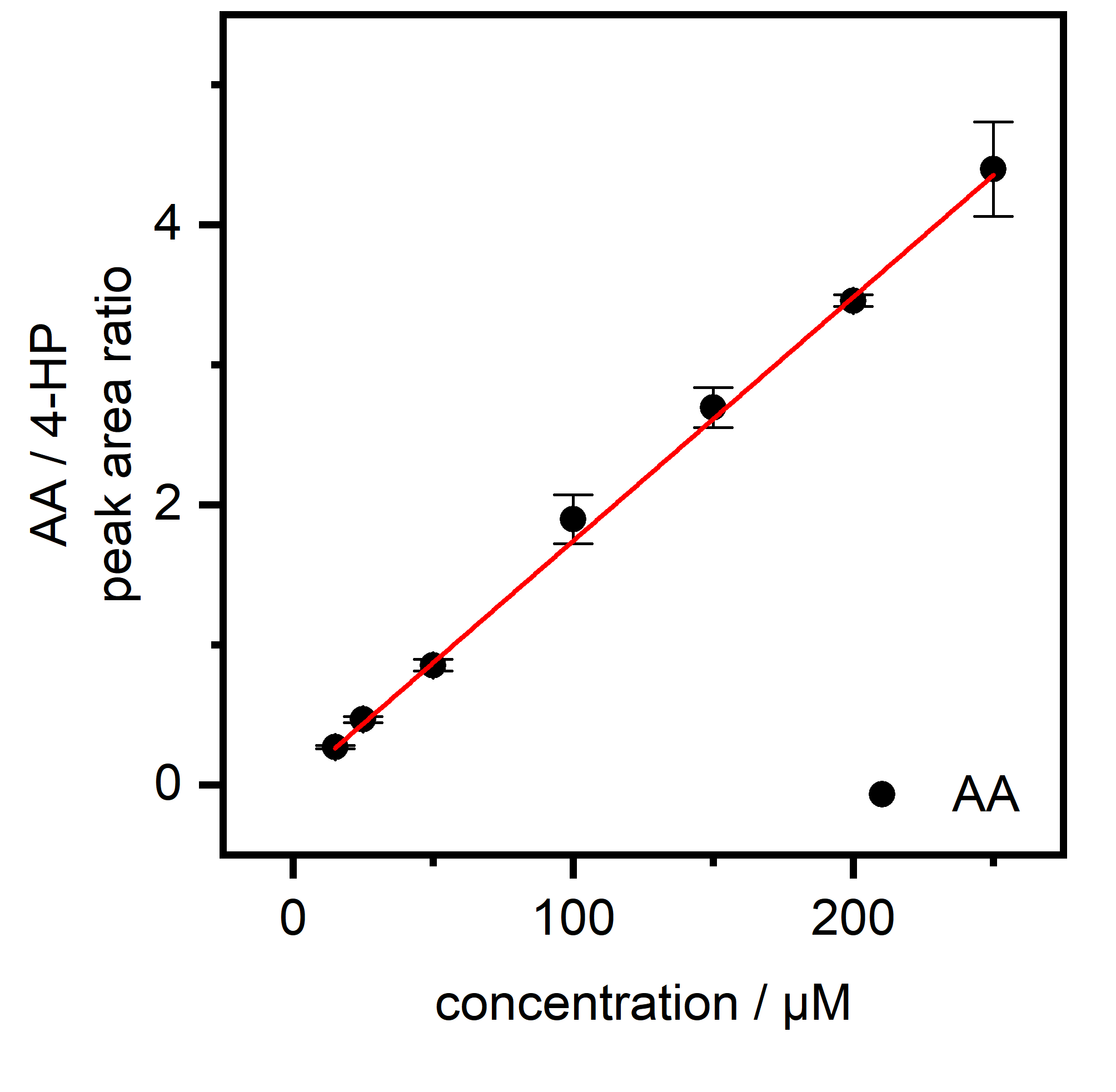


Supplementary Figure 3: Calibration plot of dialanine (AA) (m = 0.01741 ± 1.48436E-4, R^2^ = 0.99956) after the electrophoretic separation of a standard mixture consisting of diglycine, alanylglycine, glycylalanine and dialanine. The calibration curve was recorded in triplicates using a conductivity detector and 4-HP as internal standard (100 µM). Bare fused silica capillary, 80 cm; BGE, AcOH (2 M); CE inlet, 30 kV; T = 25 °C, sample injection using 30 mbar pressure for 10 s.


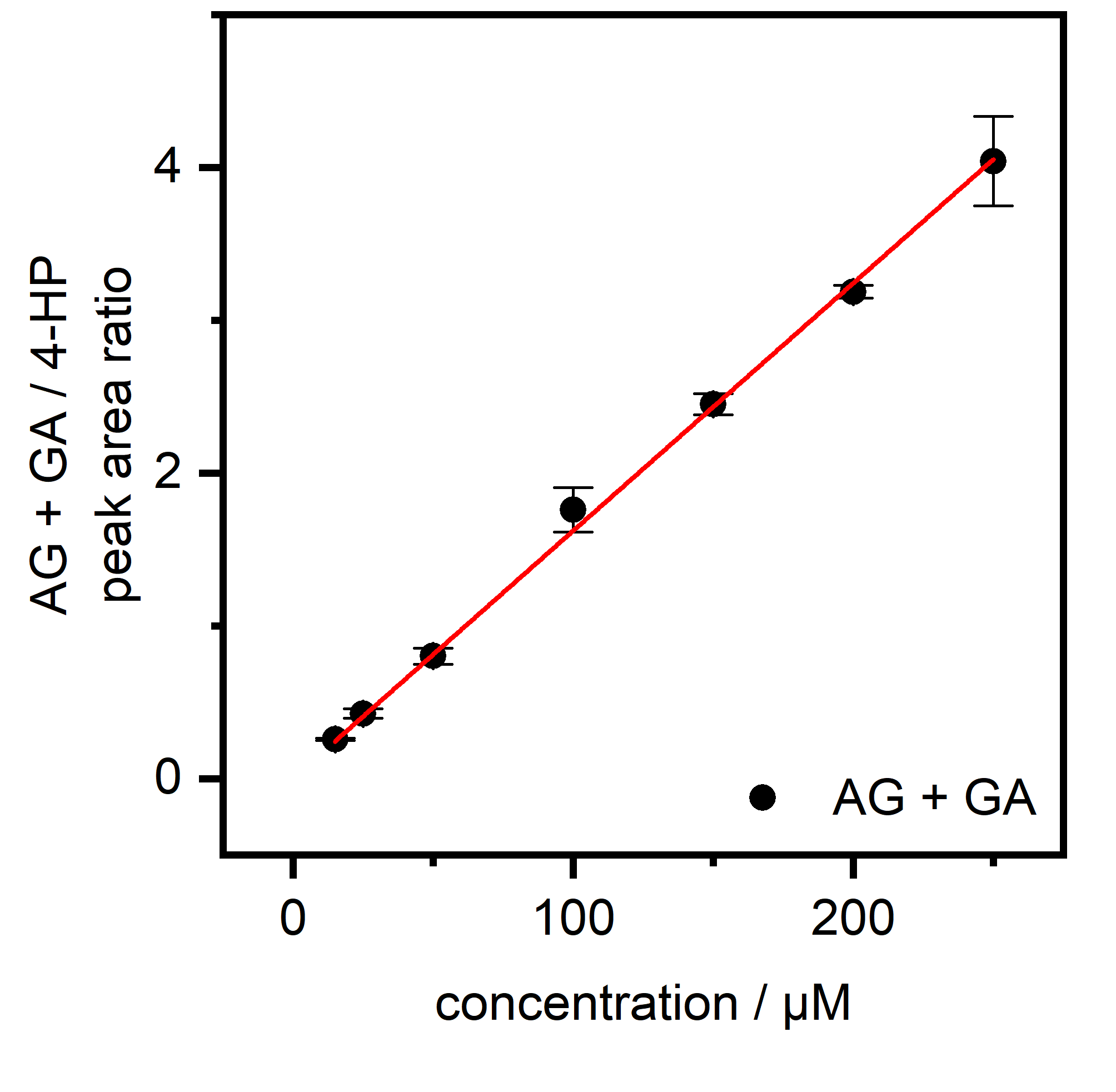


Supplementary Figure 4: Calibration plot of alanylglycine (AG) and glycylalanine (GA) (m = 0.01621 ± 1.8585E-4, R^2^ = 0.99921) after the electrophoretic separation of a standard mixture consisting of GG, AG, GA and AA. The calibration curve was recorded in triplicates using a conductivity detector and 4-HP as internal standard (100 µM). Bare fused silica capillary, 80 cm; BGE, AcOH (2 M); CE inlet, 30 kV; T = 25 °C, sample injection using 30 mbar pressure for 10 s.

## Peptide formation in SO_2_

### 400 mM, 21 d

| *n*_reactant_ = 1.2 mmol | *T* = room temperature |
| --- | --- |
| *V*_SO2_ = 3 mL | *t* = 21 d |

Supplementary Table 1: First analysis run of the copper(II)-catalysed peptide condensation in SO_2_ after 21 d with an initial reactant concentration of 400 mM.

|  | **Integral**  [10^-4^ a.u.·min] | **Dipeptide / 4-HP**  Peak area ratio | **c_sample vial_**  [µM] | **c_reaction mixture_**  [mM] | **Yield**  [%] |
| --- | --- | --- | --- | --- | --- |
| **GG** | 0.45 | 1.21 | 83.8 | 3.35 | 1.68 |
| **AG + GA** | 1.33 | 3.60 | 222 | 8.89 | 4.45 |
| **AA** | 1.33 | 3.59 | 206 | 8.25 | 4.12 |
| **4-HP** | 0.37 |  |  |  |  |

Supplementary Table 2: Second analysis run of the copper(II)-catalysed peptide condensation in SO_2_ after 21 d with an initial reactant concentration of 400 mM.

|  | **Integral**  [10^-4^ a.u.·min] | **Dipeptide / 4-HP**  Peak area ratio | **c_sample vial_**  [µM] | **c_reaction mixture_**  [mM] | **Yield**  [%] | **Ø Yield**  [%] |
| --- | --- | --- | --- | --- | --- | --- |
| **GG** | 0.46 | 1.21 | 84.1 | 3.36 | 1.68 | 1.68 ±0.01 |
| **AG + GA** | 1.31 | 3.46 | 214 | 8.54 | 4.27 | 4.35 ± 0.13 |
| **AA** | 1.32 | 3.48 | 200 | 7.99 | 4.00 | 4.06 ± 0.10 |
| **4-HP** | 0.38 |  |  |  |  |  |


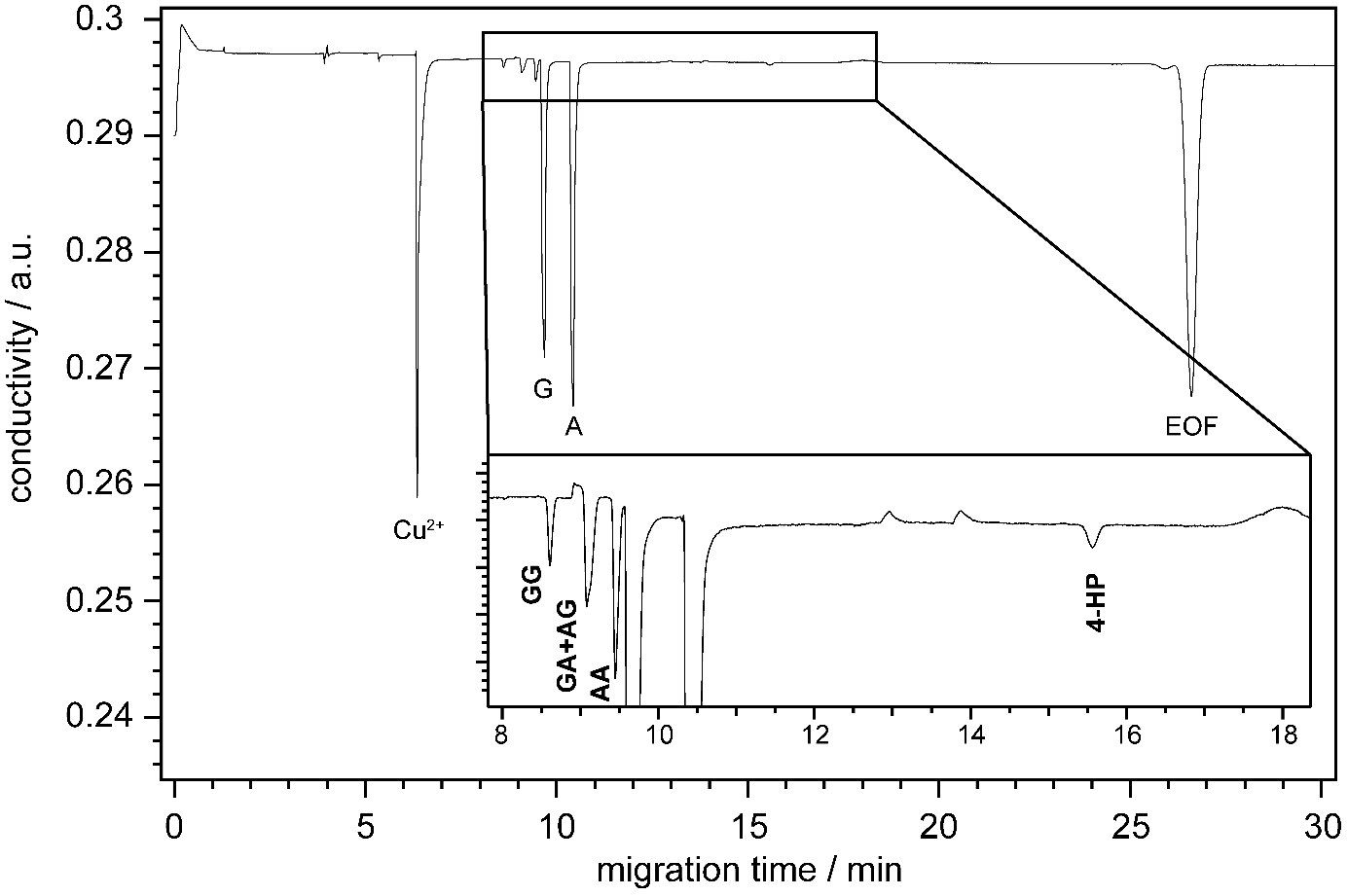


Supplementary Figure 5: First analysis run.


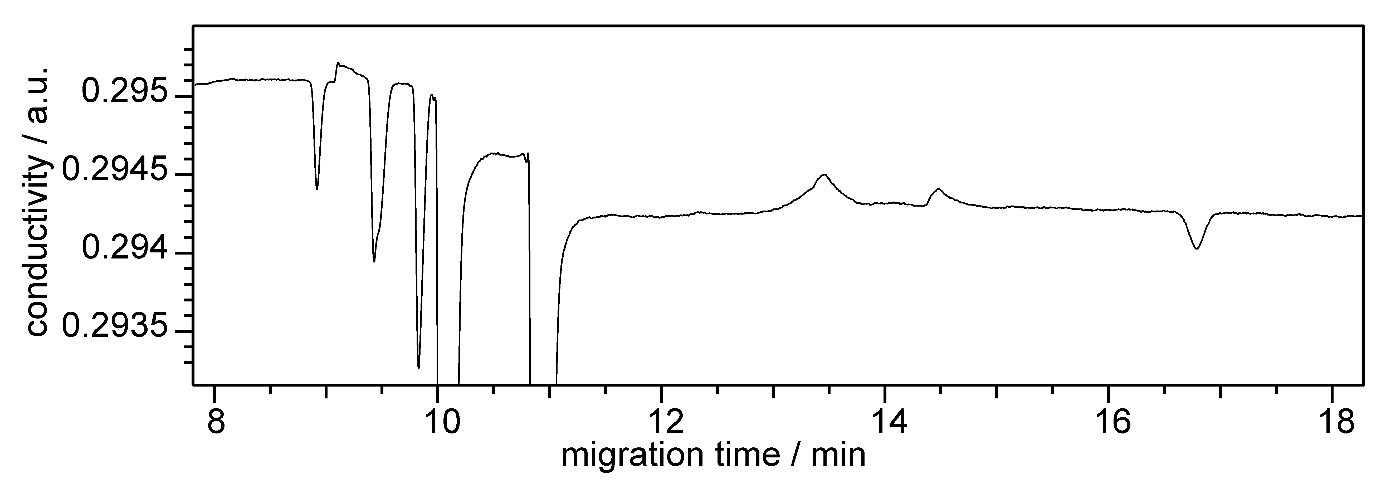


Supplementary Figure 6: Second analysis run.

### 400 mM, 7 d

| *n*_reactant_ = 1.2 mmol | *T* = room temperature |
| --- | --- |
| *V*_SO2_ = 3 mL | *t* = 7 d |

Supplementary Table 3: First analysis run of the copper(II)-catalysed peptide condensation in SO_2_ after 7 d with an initial reactant concentration of 400 mM.

|  | **Integral**  [10^-5^ a.u.·min] | **Dipeptide / 4-HP**  Peak area ratio | **c_sample vial_**  [µM] | **c_reaction mixture_**  [mM] | **Yield**  [%] |
| --- | --- | --- | --- | --- | --- |
| **GG** | 4.07 | 1.12 | 77.7 | 3.11 | 1.55 |
| **AG + GA** | 6.52 | 1.80 | 111 | 4.43 | 2.22 |
| **AA** | 3.82 | 1.05 | 60.5 | 2.42 | 1.21 |
| **4-HP** | 3.63 |  |  |  |  |

Supplementary Table 4: Second analysis run of the copper(II)-catalysed peptide condensation in SO_2_ after 7 d with an initial reactant concentration of 400 mM.

|  | **Integral**  [10^-5^ a.u.·min] | **Dipeptide / 4-HP**  Peak area ratio | **c_sample vial_**  [µM] | **c_reaction mixture_**  [mM] | **Yield**  [%] | **Ø Yield**  [%] |
| --- | --- | --- | --- | --- | --- | --- |
| **GG** | 3.96 | 1.06 | 73.3 | 2.93 | 1.47 | 1.51 ± 0.06 |
| **AG + GA** | 6.51 | 1.74 | 107 | 4.30 | 2.15 | 2.18 ± 0.05 |
| **AA** | 4.08 | 1.09 | 62.7 | 2.51 | 1.25 | 1.23 ± 0.03 |
| **4-HP** | 3.74 |  |  |  |  |  |


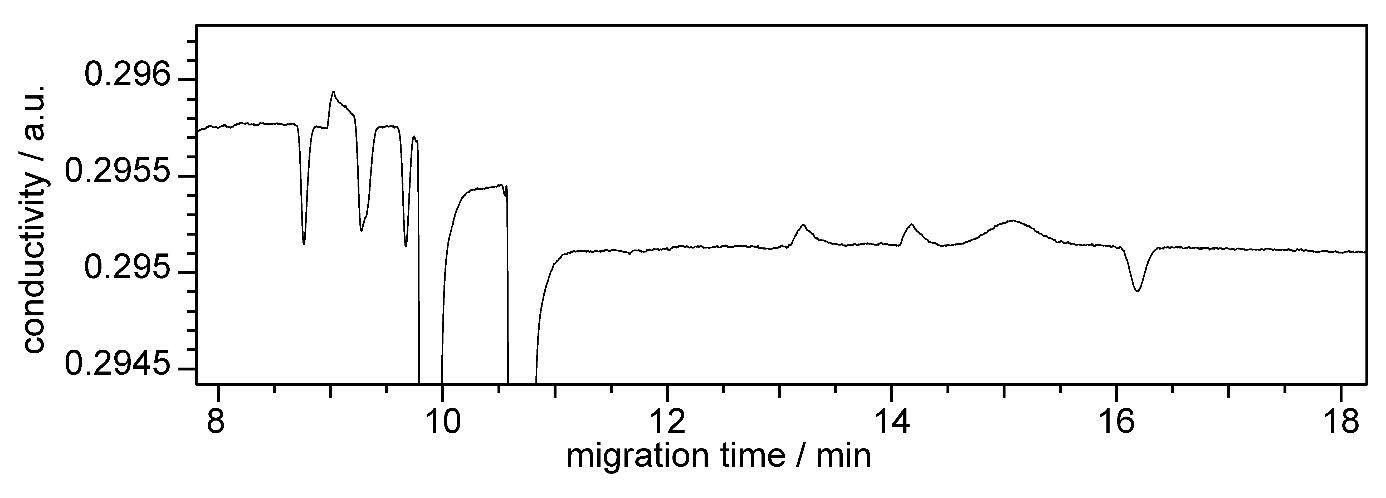


Supplementary Figure 7: First analysis run.


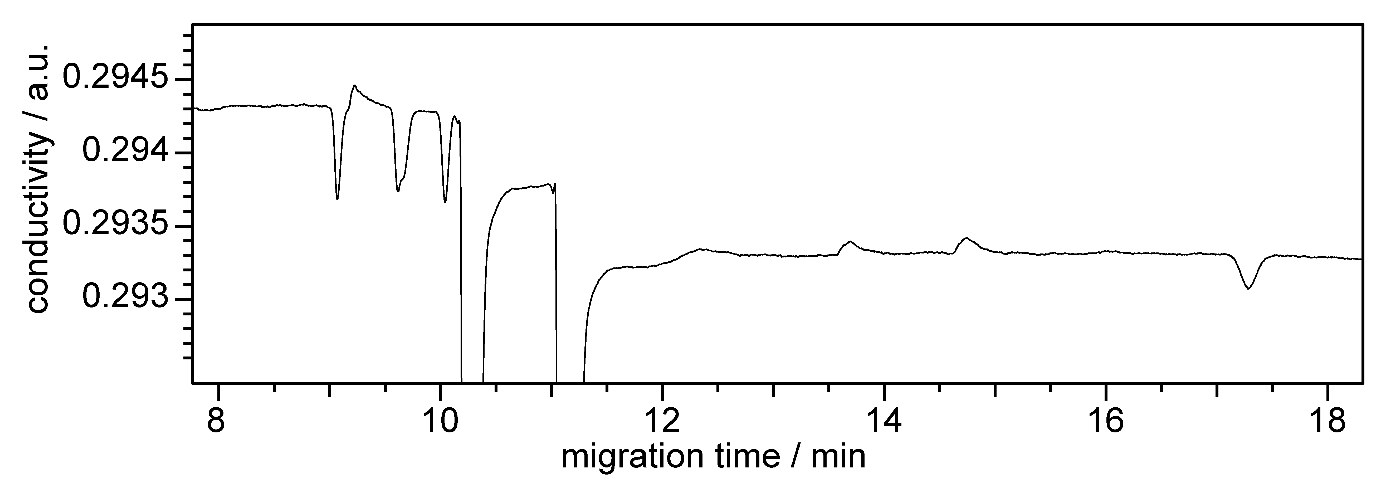


Supplementary Figure 8: Second analysis run.

### 400 mM, 3 d

| *n*_reactant_ = 1 mmol | *T* = room temperature |
| --- | --- |
| *V*_SO2_ = 2.5 mL | *t* = 3 d |

Supplementary Table 5: First analysis run of the copper(II)-catalysed peptide condensation in SO_2_ after 3 d with an initial reactant concentration of 400 mM.

|  | **Integral**  [10^-5^ a.u.·min] | **Dipeptide / 4-HP**  Peak area ratio | **c_sample vial_**  [µM] | **c_reaction mixture_**  [mM] | **Yield**  [%] |
| --- | --- | --- | --- | --- | --- |
| **GG** | 4.78 | 1.15 | 80.0 | 3.20 | 1.60 |
| **AG + GA** | 7.13 | 1.72 | 106 | 4.25 | 2.12 |
| **AA** | 3.56 | 0.86 | 49.4 | 1.98 | 0.99 |
| **4-HP** | 4.14 |  |  |  |  |

Supplementary Table 6: Second analysis run of the copper(II)-catalysed peptide condensation in SO_2_ after 3 d with an initial reactant concentration of 400 mM.

|  | **Integral**  [10^-5^ a.u.·min] | **Dipeptide / 4-HP**  Peak area ratio | **c_sample vial_**  [µM] | **c_reaction mixture_**  [mM] | **Yield**  [%] | **Ø Yield**  [%] |
| --- | --- | --- | --- | --- | --- | --- |
| **GG** | 4.49 | 1.12 | 78.0 | 3.12 | 1.56 | 1.58 ± 0.03 |
| **AG + GA** | 6.98 | 1.75 | 108 | 4.31 | 2.16 | 2.14 ± 0.03 |
| **AA** | 3.79 | 0.95 | 54.4 | 2.18 | 1.09 | 1.04 ± 0.07 |
| **4-HP** | 3.99 |  |  |  |  |  |


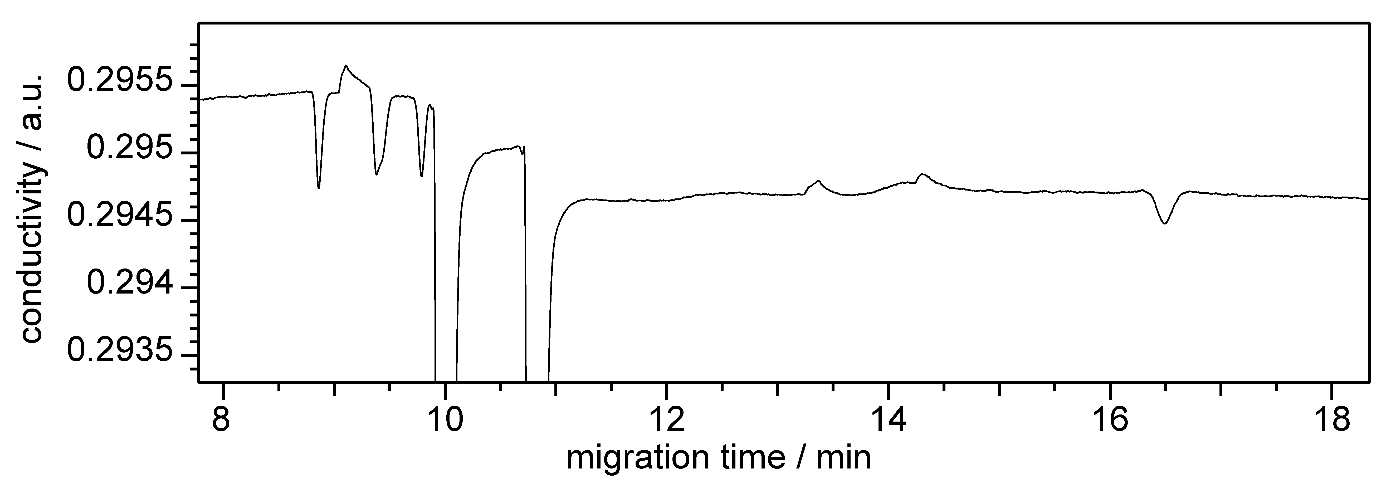


Supplementary Figure 9: First analysis run.


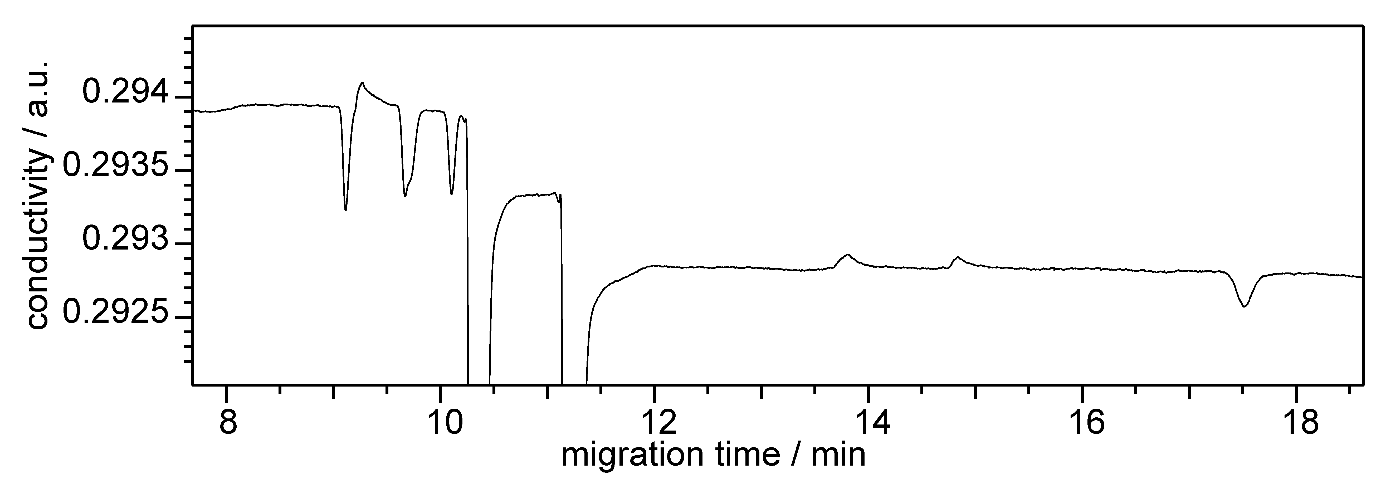


Supplementary Figure 10: Second analysis run.

### 400 mM, 1 d

| *n*_reactant_ = 1 mmol | *T* = room temperature |
| --- | --- |
| *V*_SO2_ = 2.5 mL | *t* = 1 d |

Supplementary Table 7: First analysis run of the copper(II)-catalysed peptide condensation in SO_2_ after 1 d with an initial reactant concentration of 400 mM.

|  | **Integral**  [10^-5^ a.u.·min] | **Dipeptide / 4-HP**  Peak area ratio | **c_sample vial_**  [µM] | **c_reaction mixture_**  [mM] | **Yield**  [%] |
| --- | --- | --- | --- | --- | --- |
| **GG** | 1.82 | 0.47 | 32.6 | 1.30 | 0.65 |
| **AG + GA** | 2.97 | 0.77 | 47.4 | 1.90 | 0.95 |
| **AA** | 1.53 | 0.40 | 22.7 | 0.91 | 0.45 |
| **4-HP** | 3.87 |  |  |  |  |

Supplementary Table 8: Second analysis run of the copper(II)-catalysed peptide condensation in SO_2_ after 1 d with an initial reactant concentration of 400 mM.

|  | **Integral**  [10^-5^ a.u.·min] | **Dipeptide / 4-HP**  Peak area ratio | **c_sample vial_**  [µM] | **c_reaction mixture_**  [mM] | **Yield**  [%] | **Ø Yield**  [%] |
| --- | --- | --- | --- | --- | --- | --- |
| **GG** | 1.71 | 0.47 | 32.3 | 1.29 | 0.65 | 0.65 ± 0.01 |
| **AG + GA** | 2.67 | 0.73 | 44.9 | 1.79 | 0.90 | 0.92 ± 0.04 |
| **AA** | 1.64 | 0.45 | 25.7 | 1.03 | 0.51 | 0.48 ± 0.04 |
| **4-HP** | 3.67 |  |  |  |  |  |


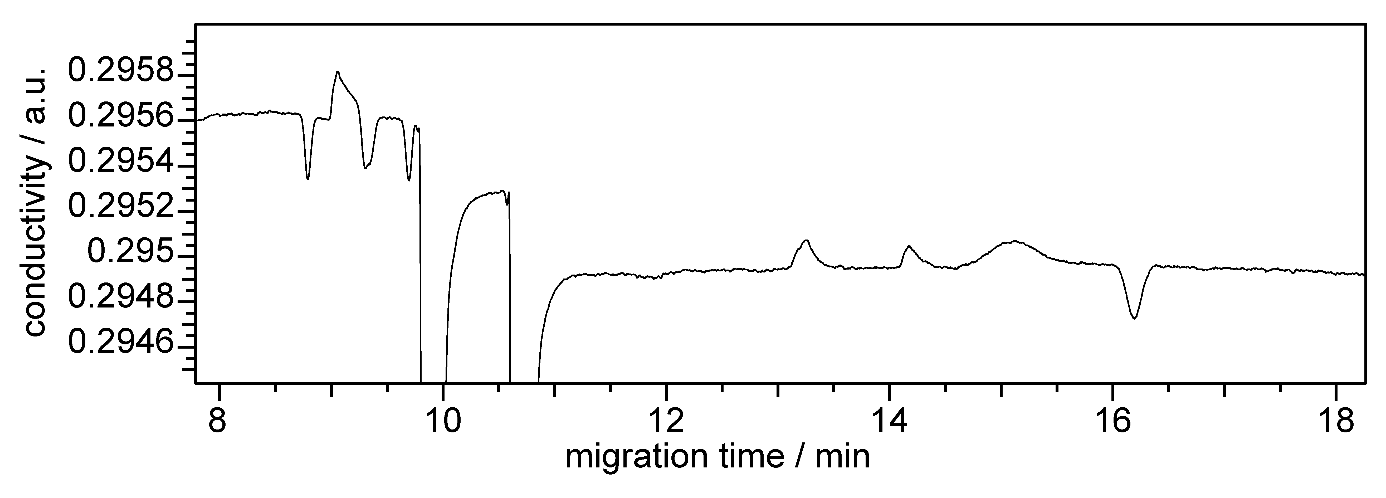


Supplementary Figure 11: First analysis run.


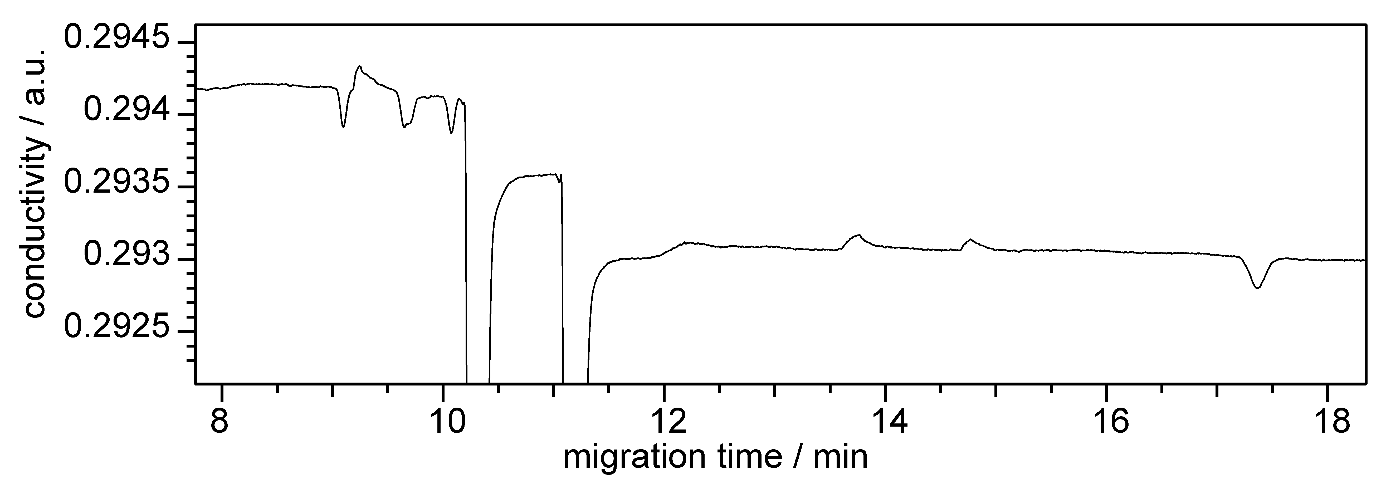


Supplementary Figure 12: Second analysis run.

### 50 mM, 7 d

| *n*_reactant_ = 75 µmol | *T* = room temperature |
| --- | --- |
| *V*_SO2_ = 1.5 mL | *t* = 7 d |

Supplementary Table 9: First analysis run of the copper(II)-catalysed peptide condensation in SO_2_ after 7 d with an initial reactant concentration of 50 mM.

|  | **Integral**  [10^-5^ a.u.·min] | **Dipeptide / 4-HP**  Peak area ratio | **c_sample vial_**  [µM] | **c_reaction mixture_**  [mM] | **Yield**  [%] |
| --- | --- | --- | --- | --- | --- |
| **GG** | 1.64 | 0.49 | 33.6 | 0.17 | 0.68 |
| **AG + GA** | 5.59 | 1.66 | 102 | 0.52 | 2.06 |
| **AA** | 9.04 | 2.67 | 154 | 0.78 | 3.10 |
| **4-HP** | 3.38 |  |  |  |  |

Supplementary Table 10: Second analysis run of the copper(II)-catalysed peptide condensation in SO_2_ after 7 d with an initial reactant concentration of 50 mM.

|  | **Integral**  [10^-5^ a.u.·min] | **Dipeptide / 4-HP**  Peak area ratio | **c_sample vial_**  [µM] | **c_reaction mixture_**  [mM] | **Yield**  [%] | **Ø Yield**  [%] |
| --- | --- | --- | --- | --- | --- | --- |
| **GG** | 1.61 | 0.40 | 28.0 | 0.14 | 0.57 | 0.62 ± 0.08 |
| **AG + GA** | 5.77 | 1.45 | 89.5 | 0.45 | 1.81 | 1.94 ± 0.18 |
| **AA** | 9.33 | 2.35 | 135 | 0.68 | 2.72 | 2.91 ± 0.27 |
| **4-HP** | 3.97 |  |  |  |  |  |


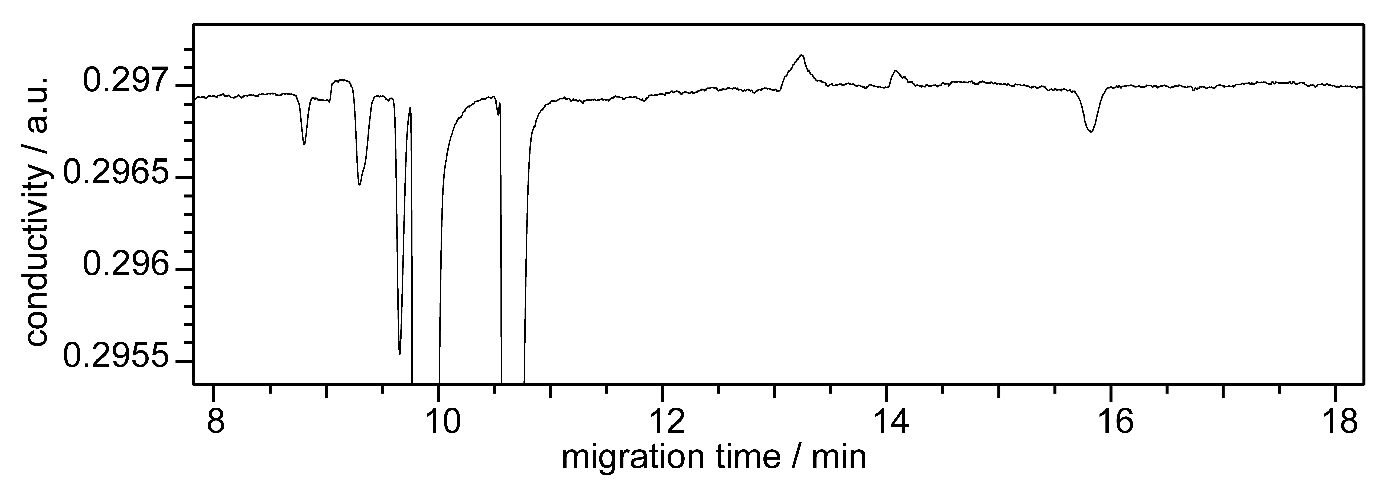


Supplementary Figure 13: First analysis run.


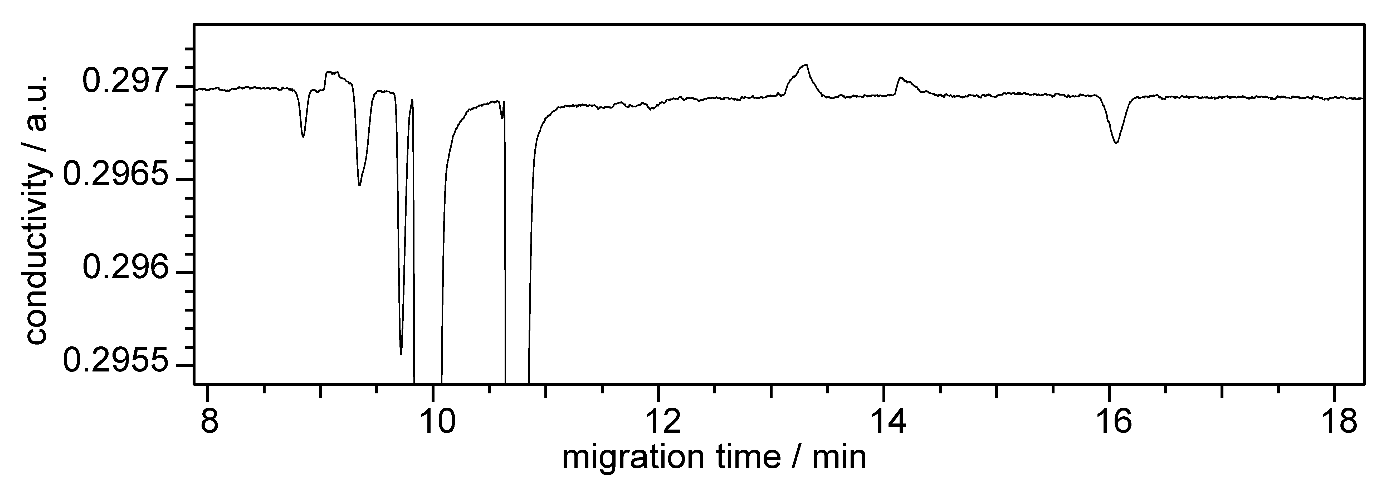


Supplementary Figure 14: Second analysis run.

### 100 mM, 7 d

| *n*_reactant_ = 300 µmol | *T* = room temperature |
| --- | --- |
| *V*_SO2_ = 3.0 mL | *t* = 7 d |

Supplementary Table 11: First analysis run of the copper(II)-catalysed peptide condensation in SO_2_ after 7 d with an initial reactant concentration of 100 mM.

|  | **Integral**  [10^-5^ a.u.·min] | **Dipeptide / 4-HP**  Peak area ratio | **c_sample vial_**  [µM] | **c_reaction mixture_**  [mM] | **Yield**  [%] |
| --- | --- | --- | --- | --- | --- |
| **GG** | 2.69 | 0.81 | 56.2 | 0.56 | 1.12 |
| **AG + GA** | 5.44 | 1.64 | 101 | 1.01 | 2.02 |
| **AA** | 4.12 | 1.24 | 71.3 | 0.71 | 1.43 |
| **4-HP** | 3.32 |  |  |  |  |

Supplementary Table 12: Second analysis run of the copper(II)-catalysed peptide condensation in SO_2_ after 7 d with an initial reactant concentration of 100 mM.

|  | **Integral**  [10^-5^ a.u.·min] | **Dipeptide / 4-HP**  Peak area ratio | **c_sample vial_**  [µM] | **c_reaction mixture_**  [mM] | **Yield**  [%] | **Ø Yield**  [%] |
| --- | --- | --- | --- | --- | --- | --- |
| **GG** | 3.18 | 0.85 | 59.0 | 0.59 | 1.18 | 1.15 ± 0.04 |
| **AG + GA** | 5.47 | 1.46 | 90.3 | 0.90 | 1.81 | 1.91 ± 0.16 |
| **AA** | 3.57 | 0.96 | 54.9 | 0.55 | 1.10 | 1.26 ± 0.23 |
| **4-HP** | 3.74 |  |  |  |  |  |


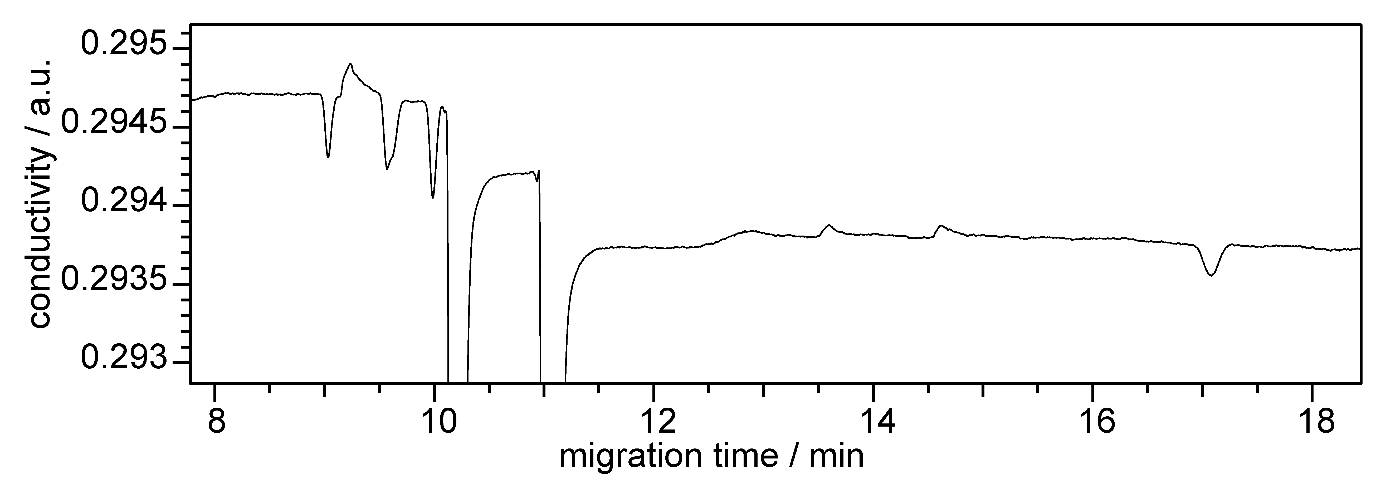


Supplementary Figure 15: First analysis run.


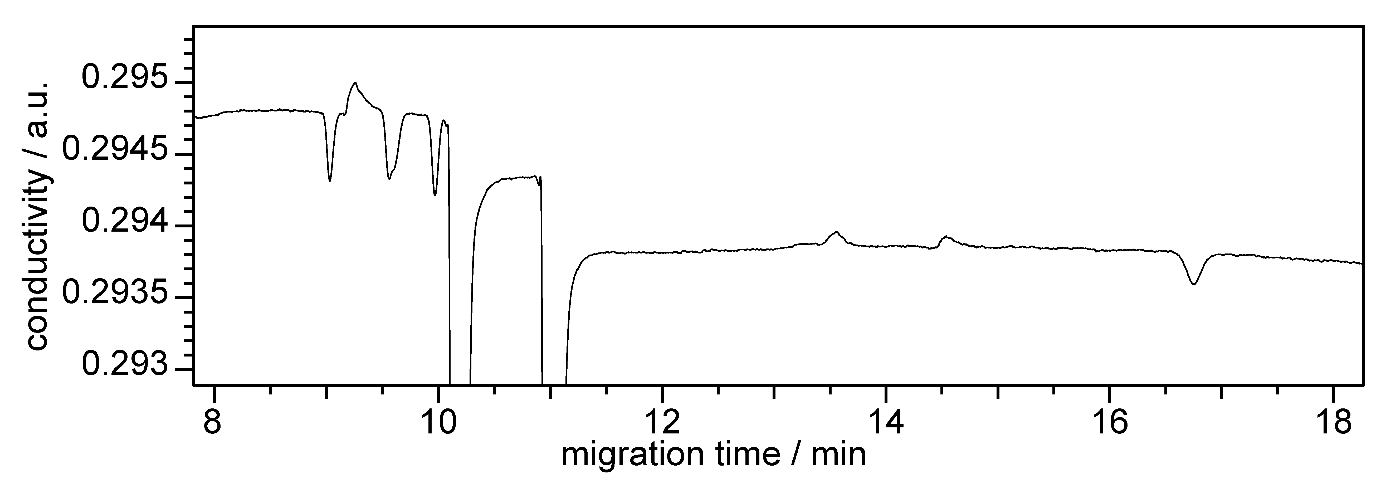


Supplementary Figure 16: Second analysis run.

### 200 mM, 7 d

| *n*_reactant_ = 300 µmol | *T* = room temperature |
| --- | --- |
| *V*_SO2_ = 1.5 mL | *t* = 7 d |

Supplementary Table 13: First analysis run of the copper(II)-catalysed peptide condensation in SO_2_ after 7 d with an initial reactant concentration of 200 mM.

|  | **Integral**  [10^-5^ a.u.·min] | **Dipeptide / 4-HP**  Peak area ratio | **c_sample vial_**  [µM] | **c_reaction mixture_**  [mM] | **Yield**  [%] |
| --- | --- | --- | --- | --- | --- |
| **GG** | 2.70 | 0.77 | 53.3 | 1.07 | 1.07 |
| **AG + GA** | 6.01 | 1.71 | 106 | 2.11 | 2.11 |
| **AA** | 5.45 | 1.55 | 89.2 | 1.78 | 1.78 |
| **4-HP** | 3.51 |  |  |  |  |

Supplementary Table 14: Second analysis run of the copper(II)-catalysed peptide condensation in SO_2_ after 7 d with an initial reactant concentration of 200 mM.

|  | **Integral**  [10^-5^ a.u.·min] | **Dipeptide / 4-HP**  Peak area ratio | **c_sample vial_**  [µM] | **c_reaction mixture_**  [mM] | **Yield**  [%] | **Ø Yield**  [%] |
| --- | --- | --- | --- | --- | --- | --- |
| **GG** | 2.58 | 0.73 | 50.3 | 1.01 | 1.01 | 1.04 ± 0.04 |
| **AG + GA** | 6.07 | 1.71 | 106 | 2.11 | 2.11 | 2.11 ± 0.02 |
| **AA** | 5.39 | 1.52 | 87.1 | 1.74 | 1.74 | 1.76 ± 0.03 |
| **4-HP** | 3.55 |  |  |  |  |  |


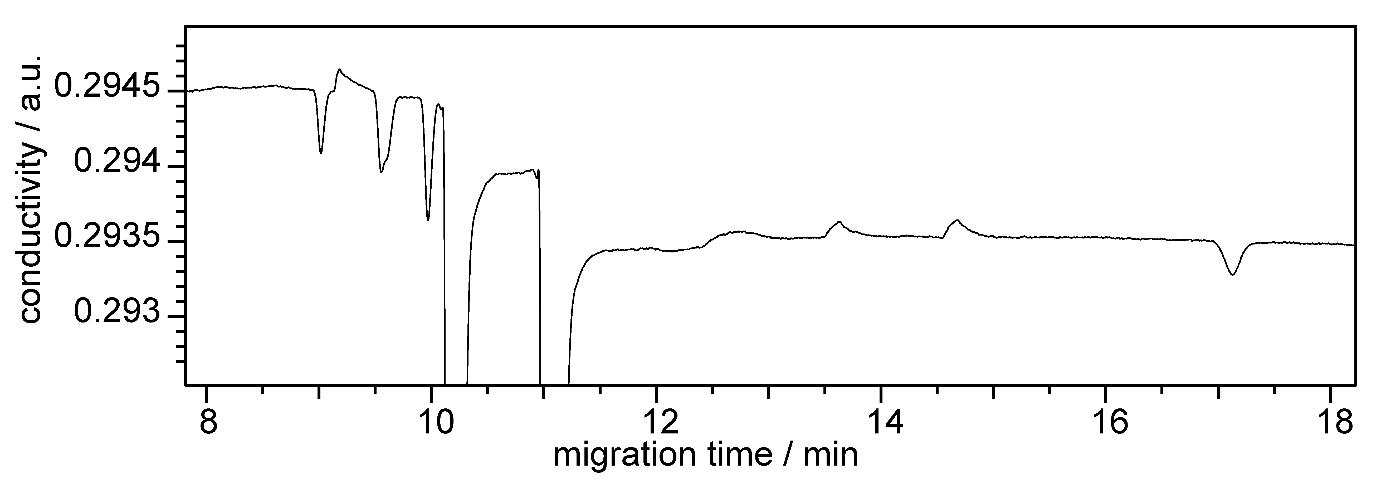


Supplementary Figure 17: First analysis run.


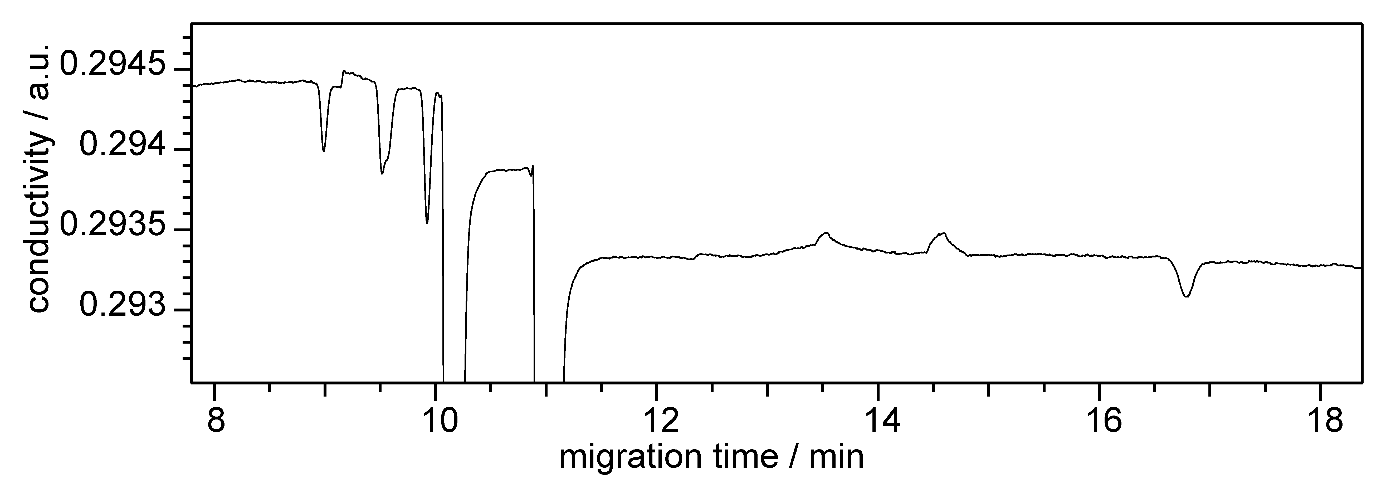


Supplementary Figure 18: Second analysis run.

# Dipeptide product spectra

|  | **C-terminus** | | |
| --- | --- | --- | --- |
|  |  | **7 d** | |
| **N-terminus** |  | **A** | **G** |
|  | **A** |  |  |
|  | **G** |  |  |

Supplementary Figure 19: Detected dipeptides of peptide condensation in SO_2_ using the Hadean mineral covellite as catalyst ( = confirmed by MS/MS, = traces, = not detected). During measurements, the concentration of alanine and glycine in the sample vial was 5 mM (instead of the usual 1 mM).

|  |  | **C-terminus** | | | | | | | | | | | | | |
| --- | --- | --- | --- | --- | --- | --- | --- | --- | --- | --- | --- | --- | --- | --- | --- |
|  |  | **7 d** | | | | | | | **21 d** | | | | | | |
|  |  | **A** | **F** | **I/L** | **M** | **P** | **V** | **W** | **A** | **F** | **I/L** | **M** | **P** | **V** | **W** |
| **N-terminus** | **A** |  |  |  |  |  |  |  |  |  |  |  |  |  |  |
|  | **F** |  |  |  |  |  |  |  |  |  |  |  |  |  |  |
|  | **I/L** |  |  |  |  |  |  |  |  |  |  |  |  |  |  |
|  | **M** |  |  |  |  |  |  |  |  |  |  |  |  |  |  |
|  | **P** |  |  |  |  |  |  |  |  |  |  |  |  |  |  |
|  | **V** |  |  |  |  |  |  |  |  |  |  |  |  |  |  |
|  | **W** |  |  |  |  |  |  |  |  |  |  |  |  |  |  |

Supplementary Figure 20: Detected dipeptides of the copper(II)-catalysed peptide condensation in SO_2_ starting from the non-polar amino acid mixture ( = confirmed by MS/MS, = traces, = not detected).

|  |  | **C-terminus** | | | | | | | | | | | | | |
| --- | --- | --- | --- | --- | --- | --- | --- | --- | --- | --- | --- | --- | --- | --- | --- |
|  |  | **7 d** | | | | | | | **21 d** | | | | | | |
|  |  | **A** | **F** | **I/L** | **M** | **P** | **V** | **W** | **A** | **F** | **I/L** | **M** | **P** | **V** | **W** |
| **N-terminus** | **A** |  |  |  |  |  |  |  |  |  |  |  |  |  |  |
|  | **F** |  |  |  |  |  |  |  |  |  |  |  |  |  |  |
|  | **I/L** |  |  |  |  |  |  |  |  |  |  |  |  |  |  |
|  | **M** |  |  |  |  |  |  |  |  |  |  |  |  |  |  |
|  | **P** |  |  |  |  |  |  |  |  |  |  |  |  |  |  |
|  | **V** |  |  |  |  |  |  |  |  |  |  |  |  |  |  |
|  | **W** |  |  |  |  |  |  |  |  |  |  |  |  |  |  |

Supplementary Figure 21: Detected dipeptides of the copper(II)-catalysed salt induced peptide formation (SIPF) in H_2_O starting from the non-polar amino acid mixture ( = confirmed by MS/MS, = traces, = not detected).

|  |  | **C-terminus** | | | | | | | | | | | | | |
| --- | --- | --- | --- | --- | --- | --- | --- | --- | --- | --- | --- | --- | --- | --- | --- |
|  |  | **7 d** | | | | | | | **21 d** | | | | | | |
|  |  | **C** | **G** | **N** | **Q** | **S** | **T** | **Y** | **C** | **G** | **N** | **Q** | **S** | **T** | **Y** |
| **N-terminus** | **C** |  |  |  |  |  |  |  |  |  |  |  |  |  |  |
|  | **G** |  |  |  |  |  |  |  |  |  |  |  |  |  |  |
|  | **N** |  |  |  |  |  |  |  |  |  |  |  |  |  |  |
|  | **Q** |  |  |  |  |  |  |  |  |  |  |  |  |  |  |
|  | **S** |  |  |  |  |  |  |  |  |  |  |  |  |  |  |
|  | **T** |  |  |  |  |  |  |  |  |  |  |  |  |  |  |
|  | **Y** |  |  |  |  |  |  |  |  |  |  |  |  |  |  |

Supplementary Figure 22: Detected dipeptides of the copper(II)-catalysed peptide condensation in SO_2_ starting from the polar, neutral amino acid mixture ( = confirmed by MS/MS, = traces, = not detected, = cystine-peptides confirmed by MS/MS, = cystine-peptides traces).

|  |  | **C-terminus** | | | | | | | | | | | | | |
| --- | --- | --- | --- | --- | --- | --- | --- | --- | --- | --- | --- | --- | --- | --- | --- |
|  |  | **7 d** | | | | | | | **21 d** | | | | | | |
|  |  | **C** | **G** | **N** | **Q** | **S** | **T** | **Y** | **C** | **G** | **N** | **Q** | **S** | **T** | **Y** |
| **N-terminus** | **C** |  |  |  |  |  |  |  |  |  |  |  |  |  |  |
|  | **G** |  |  |  |  |  |  |  |  |  |  |  |  |  |  |
|  | **N** |  |  |  |  |  |  |  |  |  |  |  |  |  |  |
|  | **Q** |  |  |  |  |  |  |  |  |  |  |  |  |  |  |
|  | **S** |  |  |  |  |  |  |  |  |  |  |  |  |  |  |
|  | **T** |  |  |  |  |  |  |  |  |  |  |  |  |  |  |
|  | **Y** |  |  |  |  |  |  |  |  |  |  |  |  |  |  |

Supplementary Figure 23: Detected dipeptides of the copper(II)-catalysed SIPF in H_2_O starting from the polar, neutral amino acid mixture ( = confirmed by MS/MS, = traces, = not detected).

|  |  | **C-terminus** | | | | | |
| --- | --- | --- | --- | --- | --- | --- | --- |
|  |  | **7 d** | | | **21 d** | | |
|  |  | **H** | **K** | **R** | **H** | **K** | **R** |
| **N-terminus** | **H** |  |  |  |  |  |  |
|  | **K** |  |  |  |  |  |  |
|  | **R** |  |  |  |  |  |  |

Supplementary Figure 24: Detected dipeptides of the copper(II)-catalysed peptide condensation in SO_2_ starting from the alkaline amino acid mixture ( = confirmed by MS/MS, = traces, = not detected).

|  |  | **C-terminus** | | | | | |
| --- | --- | --- | --- | --- | --- | --- | --- |
|  |  | **7 d** | | | **21 d** | | |
|  |  | **H** | **K** | **R** | **H** | **K** | **R** |
| **N-terminus** | **H** |  |  |  |  |  |  |
|  | **K** |  |  |  |  |  |  |
|  | **R** |  |  |  |  |  |  |

Supplementary Figure 25: Detected dipeptides of the copper(II)-catalysed SIPF in H_2_O starting from the alkaline amino acid mixture ( = confirmed by MS/MS, = traces, = not detected).

|  |  | **C-terminus** | | | |
| --- | --- | --- | --- | --- | --- |
|  |  | **7 d** | | **21 d** | |
| **N-terminus** |  | **D** | **E** | **D** | **E** |
|  | **D** |  |  |  |  |
|  | **E** |  |  |  |  |

Supplementary Figure 26: Detected dipeptides of the copper(II)-catalysed peptide condensation in SO_2_ starting from the acidic amino acid mixture ( = confirmed by MS/MS, = traces, = not detected).

|  |  | **C-terminus** | | | |
| --- | --- | --- | --- | --- | --- |
|  |  | **7 d** | | **21 d** | |
| **N-terminus** |  | **D** | **E** | **D** | **E** |
|  | **D** |  |  |  |  |
|  | **E** |  |  |  |  |

Supplementary Figure 27: Detected dipeptides of the copper(II)-catalysed SIPF in H_2_O starting from the acidic amino acid mixture ( = confirmed by MS/MS, = traces, = not detected).

|  |  | | **C-terminus** | | | | | | | | | | | | | | | | | | |
| --- | --- | --- | --- | --- | --- | --- | --- | --- | --- | --- | --- | --- | --- | --- | --- | --- | --- | --- | --- | --- | --- |
|  |  | | **7 d** | | | | | | | | | | **21 d** | | | | | | | | |
|  |  | **A** | | **D** | **E** | **G** | **I/L** | **P** | **S** | **T** | **V** | **A** | | **D** | **E** | **G** | **I/L** | **P** | **S** | **T** | **V** |
| **N-terminus** | **A** |  | |  |  |  |  |  |  |  |  |  | |  |  |  |  |  |  |  |  |
|  | **D** |  | |  |  |  |  |  |  |  |  |  | |  |  |  |  |  |  |  |  |
|  | **E** |  | |  |  |  |  |  |  |  |  |  | |  |  |  |  |  |  |  |  |
|  | **G** |  | |  |  |  |  |  |  |  |  |  | |  |  |  |  |  |  |  |  |
|  | **I/L** |  | |  |  |  |  |  |  |  |  |  | |  |  |  |  |  |  |  |  |
|  | **P** |  | |  |  |  |  |  |  |  |  |  | |  |  |  |  |  |  |  |  |
|  | **S** |  | |  |  |  |  |  |  |  |  |  | |  |  |  |  |  |  |  |  |
|  | **T** |  | |  |  |  |  |  |  |  |  |  | |  |  |  |  |  |  |  |  |
|  | **V** |  | |  |  |  |  |  |  |  |  |  | |  |  |  |  |  |  |  |  |

Supplementary Figure 28: Detected dipeptides of the copper(II)-catalysed peptide condensation in SO_2_ starting from the prebiotic amino acid mixture ( = confirmed by MS/MS, = traces, = not detected).

|  |  | | **C-terminus** | | | | | | | | | | | | | | | | | | |
| --- | --- | --- | --- | --- | --- | --- | --- | --- | --- | --- | --- | --- | --- | --- | --- | --- | --- | --- | --- | --- | --- |
|  |  | | **7 d** | | | | | | | | | | **21 d** | | | | | | | | |
|  |  | **A** | | **D** | **E** | **G** | **I/L** | **P** | **S** | **T** | **V** | **A** | | **D** | **E** | **G** | **I/L** | **P** | **S** | **T** | **V** |
| **N-terminus** | **A** |  | |  |  |  |  |  |  |  |  |  | |  |  |  |  |  |  |  |  |
|  | **D** |  | |  |  |  |  |  |  |  |  |  | |  |  |  |  |  |  |  |  |
|  | **E** |  | |  |  |  |  |  |  |  |  |  | |  |  |  |  |  |  |  |  |
|  | **G** |  | |  |  |  |  |  |  |  |  |  | |  |  |  |  |  |  |  |  |
|  | **I/L** |  | |  |  |  |  |  |  |  |  |  | |  |  |  |  |  |  |  |  |
|  | **P** |  | |  |  |  |  |  |  |  |  |  | |  |  |  |  |  |  |  |  |
|  | **S** |  | |  |  |  |  |  |  |  |  |  | |  |  |  |  |  |  |  |  |
|  | **T** |  | |  |  |  |  |  |  |  |  |  | |  |  |  |  |  |  |  |  |
|  | **V** |  | |  |  |  |  |  |  |  |  |  | |  |  |  |  |  |  |  |  |

Supplementary Figure 29: Detected dipeptides of the copper(II)-catalysed SIPF in H_2_O starting from the prebiotic amino acid mixture ( = confirmed by MS/MS, = traces, = not detected).

|  |  | | **C-terminus** | | | | | | | | | | | | | | | | | | | |
| --- | --- | --- | --- | --- | --- | --- | --- | --- | --- | --- | --- | --- | --- | --- | --- | --- | --- | --- | --- | --- | --- | --- |
|  |  | **A** | | **F** | **I/L** | **M** | **P** | **V** | **W** | **C** | **G** | **N** | **Q** | **S** | **T** | **Y** | **H** | **K** | **R** | **D** | **E** |  |
| **N-terminus** | **A** |  | |  |  |  |  |  |  |  |  |  |  |  |  |  |  |  |  |  |  |  |
|  | **F** |  | |  |  |  |  |  |  |  |  |  |  |  |  |  |  |  |  |  |  |  |
|  | **I/L** |  | |  |  |  |  |  |  |  |  |  |  |  |  |  |  |  |  |  |  |  |
|  | **M** |  | |  |  |  |  |  |  |  |  |  |  |  |  |  |  |  |  |  |  |  |
|  | **P** |  | |  |  |  |  |  |  |  |  |  |  |  |  |  |  |  |  |  |  |  |
|  | **V** |  | |  |  |  |  |  |  |  |  |  |  |  |  |  |  |  |  |  |  |  |
|  | **W** |  | |  |  |  |  |  |  |  |  |  |  |  |  |  |  |  |  |  |  |  |
|  | **C** |  | |  |  |  |  |  |  |  |  |  |  |  |  |  |  |  |  |  |  |  |
|  | **G** |  | |  |  |  |  |  |  |  |  |  |  |  |  |  |  |  |  |  |  |  |
|  | **N** |  | |  |  |  |  |  |  |  |  |  |  |  |  |  |  |  |  |  |  |  |
|  | **Q** |  | |  |  |  |  |  |  |  |  |  |  |  |  |  |  |  |  |  |  |  |
|  | **S** |  | |  |  |  |  |  |  |  |  |  |  |  |  |  |  |  |  |  |  |  |
|  | **T** |  | |  |  |  |  |  |  |  |  |  |  |  |  |  |  |  |  |  |  |  |
|  | **Y** |  | |  |  |  |  |  |  |  |  |  |  |  |  |  |  |  |  |  |  |  |
|  | **H** |  | |  |  |  |  |  |  |  |  |  |  |  |  |  |  |  |  |  |  |  |
|  | **K** |  | |  |  |  |  |  |  |  |  |  |  |  |  |  |  |  |  |  |  |  |
|  | **R** |  | |  |  |  |  |  |  |  |  |  |  |  |  |  |  |  |  |  |  |  |
|  | **D** |  | |  |  |  |  |  |  |  |  |  |  |  |  |  |  |  |  |  |  |  |
|  | **E** |  | |  |  |  |  |  |  |  |  |  |  |  |  |  |  |  |  |  |  |  |

Supplementary Figure 30: Detected dipeptides of the copper(II)-catalysed peptide condensation in SO_2_ after 7 d starting from the full amino acid mixture ( = confirmed by MS/MS, = traces, = not detected).

|  |  | | **C-terminus** | | | | | | | | | | | | | | | | | | | |
| --- | --- | --- | --- | --- | --- | --- | --- | --- | --- | --- | --- | --- | --- | --- | --- | --- | --- | --- | --- | --- | --- | --- |
|  |  | **A** | | **F** | **I/L** | **M** | **P** | **V** | **W** | **C** | **G** | **N** | **Q** | **S** | **T** | **Y** | **H** | **K** | **R** | **D** | **E** |  |
| **N-terminus** | **A** |  | |  |  |  |  |  |  |  |  |  |  |  |  |  |  |  |  |  |  |  |
|  | **F** |  | |  |  |  |  |  |  |  |  |  |  |  |  |  |  |  |  |  |  |  |
|  | **I/L** |  | |  |  |  |  |  |  |  |  |  |  |  |  |  |  |  |  |  |  |  |
|  | **M** |  | |  |  |  |  |  |  |  |  |  |  |  |  |  |  |  |  |  |  |  |
|  | **P** |  | |  |  |  |  |  |  |  |  |  |  |  |  |  |  |  |  |  |  |  |
|  | **V** |  | |  |  |  |  |  |  |  |  |  |  |  |  |  |  |  |  |  |  |  |
|  | **W** |  | |  |  |  |  |  |  |  |  |  |  |  |  |  |  |  |  |  |  |  |
|  | **C** |  | |  |  |  |  |  |  |  |  |  |  |  |  |  |  |  |  |  |  |  |
|  | **G** |  | |  |  |  |  |  |  |  |  |  |  |  |  |  |  |  |  |  |  |  |
|  | **N** |  | |  |  |  |  |  |  |  |  |  |  |  |  |  |  |  |  |  |  |  |
|  | **Q** |  | |  |  |  |  |  |  |  |  |  |  |  |  |  |  |  |  |  |  |  |
|  | **S** |  | |  |  |  |  |  |  |  |  |  |  |  |  |  |  |  |  |  |  |  |
|  | **T** |  | |  |  |  |  |  |  |  |  |  |  |  |  |  |  |  |  |  |  |  |
|  | **Y** |  | |  |  |  |  |  |  |  |  |  |  |  |  |  |  |  |  |  |  |  |
|  | **H** |  | |  |  |  |  |  |  |  |  |  |  |  |  |  |  |  |  |  |  |  |
|  | **K** |  | |  |  |  |  |  |  |  |  |  |  |  |  |  |  |  |  |  |  |  |
|  | **R** |  | |  |  |  |  |  |  |  |  |  |  |  |  |  |  |  |  |  |  |  |
|  | **D** |  | |  |  |  |  |  |  |  |  |  |  |  |  |  |  |  |  |  |  |  |
|  | **E** |  | |  |  |  |  |  |  |  |  |  |  |  |  |  |  |  |  |  |  |  |

Supplementary Figure 31: Detected dipeptides of the copper(II)-catalysed peptide condensation in SO_2_ after 21 d starting from the full amino acid mixture ( = confirmed by MS/MS, = traces, = not detected, = cysteine-peptide traces).

|  |  | | **C-terminus** | | | | | | | | | | | | | | | | | | | |
| --- | --- | --- | --- | --- | --- | --- | --- | --- | --- | --- | --- | --- | --- | --- | --- | --- | --- | --- | --- | --- | --- | --- |
|  |  | **A** | | **F** | **I/L** | **M** | **P** | **V** | **W** | **C** | **G** | **N** | **Q** | **S** | **T** | **Y** | **H** | **K** | **R** | **D** | **E** |  |
| **N-terminus** | **A** |  | |  |  |  |  |  |  |  |  |  |  |  |  |  |  |  |  |  |  |  |
|  | **F** |  | |  |  |  |  |  |  |  |  |  |  |  |  |  |  |  |  |  |  |  |
|  | **I/L** |  | |  |  |  |  |  |  |  |  |  |  |  |  |  |  |  |  |  |  |  |
|  | **M** |  | |  |  |  |  |  |  |  |  |  |  |  |  |  |  |  |  |  |  |  |
|  | **P** |  | |  |  |  |  |  |  |  |  |  |  |  |  |  |  |  |  |  |  |  |
|  | **V** |  | |  |  |  |  |  |  |  |  |  |  |  |  |  |  |  |  |  |  |  |
|  | **W** |  | |  |  |  |  |  |  |  |  |  |  |  |  |  |  |  |  |  |  |  |
|  | **C** |  | |  |  |  |  |  |  |  |  |  |  |  |  |  |  |  |  |  |  |  |
|  | **G** |  | |  |  |  |  |  |  |  |  |  |  |  |  |  |  |  |  |  |  |  |
|  | **N** |  | |  |  |  |  |  |  |  |  |  |  |  |  |  |  |  |  |  |  |  |
|  | **Q** |  | |  |  |  |  |  |  |  |  |  |  |  |  |  |  |  |  |  |  |  |
|  | **S** |  | |  |  |  |  |  |  |  |  |  |  |  |  |  |  |  |  |  |  |  |
|  | **T** |  | |  |  |  |  |  |  |  |  |  |  |  |  |  |  |  |  |  |  |  |
|  | **Y** |  | |  |  |  |  |  |  |  |  |  |  |  |  |  |  |  |  |  |  |  |
|  | **H** |  | |  |  |  |  |  |  |  |  |  |  |  |  |  |  |  |  |  |  |  |
|  | **K** |  | |  |  |  |  |  |  |  |  |  |  |  |  |  |  |  |  |  |  |  |
|  | **R** |  | |  |  |  |  |  |  |  |  |  |  |  |  |  |  |  |  |  |  |  |
|  | **D** |  | |  |  |  |  |  |  |  |  |  |  |  |  |  |  |  |  |  |  |  |
|  | **E** |  | |  |  |  |  |  |  |  |  |  |  |  |  |  |  |  |  |  |  |  |

Supplementary Figure 32: Detected dipeptides of the copper(II)-catalysed SIPF in H_2_O after 7 d starting from the full amino acid mixture ( = confirmed by MS/MS, = traces, = not detected).

|  |  | | **C-terminus** | | | | | | | | | | | | | | | | | | | |
| --- | --- | --- | --- | --- | --- | --- | --- | --- | --- | --- | --- | --- | --- | --- | --- | --- | --- | --- | --- | --- | --- | --- |
|  |  | **A** | | **F** | **I/L** | **M** | **P** | **V** | **W** | **C** | **G** | **N** | **Q** | **S** | **T** | **Y** | **H** | **K** | **R** | **D** | **E** |  |
| **N-terminus** | **A** |  | |  |  |  |  |  |  |  |  |  |  |  |  |  |  |  |  |  |  |  |
|  | **F** |  | |  |  |  |  |  |  |  |  |  |  |  |  |  |  |  |  |  |  |  |
|  | **I/L** |  | |  |  |  |  |  |  |  |  |  |  |  |  |  |  |  |  |  |  |  |
|  | **M** |  | |  |  |  |  |  |  |  |  |  |  |  |  |  |  |  |  |  |  |  |
|  | **P** |  | |  |  |  |  |  |  |  |  |  |  |  |  |  |  |  |  |  |  |  |
|  | **V** |  | |  |  |  |  |  |  |  |  |  |  |  |  |  |  |  |  |  |  |  |
|  | **W** |  | |  |  |  |  |  |  |  |  |  |  |  |  |  |  |  |  |  |  |  |
|  | **C** |  | |  |  |  |  |  |  |  |  |  |  |  |  |  |  |  |  |  |  |  |
|  | **G** |  | |  |  |  |  |  |  |  |  |  |  |  |  |  |  |  |  |  |  |  |
|  | **N** |  | |  |  |  |  |  |  |  |  |  |  |  |  |  |  |  |  |  |  |  |
|  | **Q** |  | |  |  |  |  |  |  |  |  |  |  |  |  |  |  |  |  |  |  |  |
|  | **S** |  | |  |  |  |  |  |  |  |  |  |  |  |  |  |  |  |  |  |  |  |
|  | **T** |  | |  |  |  |  |  |  |  |  |  |  |  |  |  |  |  |  |  |  |  |
|  | **Y** |  | |  |  |  |  |  |  |  |  |  |  |  |  |  |  |  |  |  |  |  |
|  | **H** |  | |  |  |  |  |  |  |  |  |  |  |  |  |  |  |  |  |  |  |  |
|  | **K** |  | |  |  |  |  |  |  |  |  |  |  |  |  |  |  |  |  |  |  |  |
|  | **R** |  | |  |  |  |  |  |  |  |  |  |  |  |  |  |  |  |  |  |  |  |
|  | **D** |  | |  |  |  |  |  |  |  |  |  |  |  |  |  |  |  |  |  |  |  |
|  | **E** |  | |  |  |  |  |  |  |  |  |  |  |  |  |  |  |  |  |  |  |  |

Supplementary Figure 33: Detected dipeptides of the copper(II)-catalysed SIPF in H_2_O after 21 d starting from the full amino acid mixture ( = confirmed by MS/MS, = traces, = not detected).

|  |  | | **C-terminus** | | | | | | | | | | | | | | | | | | | |
| --- | --- | --- | --- | --- | --- | --- | --- | --- | --- | --- | --- | --- | --- | --- | --- | --- | --- | --- | --- | --- | --- | --- |
|  |  | **A** | | **F** | **I/L** | **M** | **P** | **V** | **W** | **C** | **G** | **N** | **Q** | **S** | **T** | **Y** | **H** | **K** | **R** | **D** | **E** |  |
| **N-terminus** | **A** |  | |  |  |  |  |  |  |  |  |  |  |  |  |  |  |  |  |  |  |  |
|  | **F** |  | |  |  |  |  |  |  |  |  |  |  |  |  |  |  |  |  |  |  |  |
|  | **I/L** |  | |  |  |  |  |  |  |  |  |  |  |  |  |  |  |  |  |  |  |  |
|  | **M** |  | |  |  |  |  |  |  |  |  |  |  |  |  |  |  |  |  |  |  |  |
|  | **P** |  | |  |  |  |  |  |  |  |  |  |  |  |  |  |  |  |  |  |  |  |
|  | **V** |  | |  |  |  |  |  |  |  |  |  |  |  |  |  |  |  |  |  |  |  |
|  | **W** |  | |  |  |  |  |  |  |  |  |  |  |  |  |  |  |  |  |  |  |  |
|  | **C** |  | |  |  |  |  |  |  |  |  |  |  |  |  |  |  |  |  |  |  |  |
|  | **G** |  | |  |  |  |  |  |  |  |  |  |  |  |  |  |  |  |  |  |  |  |
|  | **N** |  | |  |  |  |  |  |  |  |  |  |  |  |  |  |  |  |  |  |  |  |
|  | **Q** |  | |  |  |  |  |  |  |  |  |  |  |  |  |  |  |  |  |  |  |  |
|  | **S** |  | |  |  |  |  |  |  |  |  |  |  |  |  |  |  |  |  |  |  |  |
|  | **T** |  | |  |  |  |  |  |  |  |  |  |  |  |  |  |  |  |  |  |  |  |
|  | **Y** |  | |  |  |  |  |  |  |  |  |  |  |  |  |  |  |  |  |  |  |  |
|  | **H** |  | |  |  |  |  |  |  |  |  |  |  |  |  |  |  |  |  |  |  |  |
|  | **K** |  | |  |  |  |  |  |  |  |  |  |  |  |  |  |  |  |  |  |  |  |
|  | **R** |  | |  |  |  |  |  |  |  |  |  |  |  |  |  |  |  |  |  |  |  |
|  | **D** |  | |  |  |  |  |  |  |  |  |  |  |  |  |  |  |  |  |  |  |  |
|  | **E** |  | |  |  |  |  |  |  |  |  |  |  |  |  |  |  |  |  |  |  |  |

Supplementary Figure 34: Detected dipeptides of the copper(II)-catalysed peptide condensation in SO_2_ after 21 d starting from the 50 mM full amino acid mixture ( = confirmed by MS/MS, = traces, = not detected).

|  |  | | **C-terminus** | | | | | | | | | | | | | | | | | | | |
| --- | --- | --- | --- | --- | --- | --- | --- | --- | --- | --- | --- | --- | --- | --- | --- | --- | --- | --- | --- | --- | --- | --- |
|  |  | **A** | | **F** | **I/L** | **M** | **P** | **V** | **W** | **C** | **G** | **N** | **Q** | **S** | **T** | **Y** | **H** | **K** | **R** | **D** | **E** |  |
| **N-terminus** | **A** |  | |  |  |  |  |  |  |  |  |  |  |  |  |  |  |  |  |  |  |  |
|  | **F** |  | |  |  |  |  |  |  |  |  |  |  |  |  |  |  |  |  |  |  |  |
|  | **I/L** |  | |  |  |  |  |  |  |  |  |  |  |  |  |  |  |  |  |  |  |  |
|  | **M** |  | |  |  |  |  |  |  |  |  |  |  |  |  |  |  |  |  |  |  |  |
|  | **P** |  | |  |  |  |  |  |  |  |  |  |  |  |  |  |  |  |  |  |  |  |
|  | **V** |  | |  |  |  |  |  |  |  |  |  |  |  |  |  |  |  |  |  |  |  |
|  | **W** |  | |  |  |  |  |  |  |  |  |  |  |  |  |  |  |  |  |  |  |  |
|  | **C** |  | |  |  |  |  |  |  |  |  |  |  |  |  |  |  |  |  |  |  |  |
|  | **G** |  | |  |  |  |  |  |  |  |  |  |  |  |  |  |  |  |  |  |  |  |
|  | **N** |  | |  |  |  |  |  |  |  |  |  |  |  |  |  |  |  |  |  |  |  |
|  | **Q** |  | |  |  |  |  |  |  |  |  |  |  |  |  |  |  |  |  |  |  |  |
|  | **S** |  | |  |  |  |  |  |  |  |  |  |  |  |  |  |  |  |  |  |  |  |
|  | **T** |  | |  |  |  |  |  |  |  |  |  |  |  |  |  |  |  |  |  |  |  |
|  | **Y** |  | |  |  |  |  |  |  |  |  |  |  |  |  |  |  |  |  |  |  |  |
|  | **H** |  | |  |  |  |  |  |  |  |  |  |  |  |  |  |  |  |  |  |  |  |
|  | **K** |  | |  |  |  |  |  |  |  |  |  |  |  |  |  |  |  |  |  |  |  |
|  | **R** |  | |  |  |  |  |  |  |  |  |  |  |  |  |  |  |  |  |  |  |  |
|  | **D** |  | |  |  |  |  |  |  |  |  |  |  |  |  |  |  |  |  |  |  |  |
|  | **E** |  | |  |  |  |  |  |  |  |  |  |  |  |  |  |  |  |  |  |  |  |

Supplementary Figure 35: Detected dipeptides of the copper(II)-catalysed SIPF in H_2_O after 7 d starting from the 50 mM full amino acid mixture ( = confirmed by MS/MS, = traces, = not detected).

|  |  | | **C-terminus** | | | | | | | | | | | | | | | | | | | |
| --- | --- | --- | --- | --- | --- | --- | --- | --- | --- | --- | --- | --- | --- | --- | --- | --- | --- | --- | --- | --- | --- | --- |
|  |  | **A** | | **F** | **I/L** | **M** | **P** | **V** | **W** | **C** | **G** | **N** | **Q** | **S** | **T** | **Y** | **H** | **K** | **R** | **D** | **E** |  |
| **N-terminus** | **A** |  | |  |  |  |  |  |  |  |  |  |  |  |  |  |  |  |  |  |  |  |
|  | **F** |  | |  |  |  |  |  |  |  |  |  |  |  |  |  |  |  |  |  |  |  |
|  | **I/L** |  | |  |  |  |  |  |  |  |  |  |  |  |  |  |  |  |  |  |  |  |
|  | **M** |  | |  |  |  |  |  |  |  |  |  |  |  |  |  |  |  |  |  |  |  |
|  | **P** |  | |  |  |  |  |  |  |  |  |  |  |  |  |  |  |  |  |  |  |  |
|  | **V** |  | |  |  |  |  |  |  |  |  |  |  |  |  |  |  |  |  |  |  |  |
|  | **W** |  | |  |  |  |  |  |  |  |  |  |  |  |  |  |  |  |  |  |  |  |
|  | **C** |  | |  |  |  |  |  |  |  |  |  |  |  |  |  |  |  |  |  |  |  |
|  | **G** |  | |  |  |  |  |  |  |  |  |  |  |  |  |  |  |  |  |  |  |  |
|  | **N** |  | |  |  |  |  |  |  |  |  |  |  |  |  |  |  |  |  |  |  |  |
|  | **Q** |  | |  |  |  |  |  |  |  |  |  |  |  |  |  |  |  |  |  |  |  |
|  | **S** |  | |  |  |  |  |  |  |  |  |  |  |  |  |  |  |  |  |  |  |  |
|  | **T** |  | |  |  |  |  |  |  |  |  |  |  |  |  |  |  |  |  |  |  |  |
|  | **Y** |  | |  |  |  |  |  |  |  |  |  |  |  |  |  |  |  |  |  |  |  |
|  | **H** |  | |  |  |  |  |  |  |  |  |  |  |  |  |  |  |  |  |  |  |  |
|  | **K** |  | |  |  |  |  |  |  |  |  |  |  |  |  |  |  |  |  |  |  |  |
|  | **R** |  | |  |  |  |  |  |  |  |  |  |  |  |  |  |  |  |  |  |  |  |
|  | **D** |  | |  |  |  |  |  |  |  |  |  |  |  |  |  |  |  |  |  |  |  |
|  | **E** |  | |  |  |  |  |  |  |  |  |  |  |  |  |  |  |  |  |  |  |  |

Supplementary Figure 36: Detected dipeptides of the copper(II)-catalysed SIPF in H_2_O after 21 d starting from the 50 mM full amino acid mixture ( = confirmed by MS/MS, = traces, = not detected).

# Extracted ion electropherograms and mass spectra

Unless stated otherwise, the following measurement conditions were applied: LPA-coated capillary, 80 cm, 50 µm inner diameter; background electrolyte (BGE), AcOH (2 M); sample injection by applying 30 mbar pressure for 10 s; assisting pressure of 30 mbar; CE inlet, 30 kV; emitter, 3.2 kV; sheath liquid, H_2_O/isopropanol (50/50) with 0.05 % formic acid; scan range: *m/z* 122-750; NCE 30 %. Drops in the ion counts are due to the simultaneously recorded data dependant MS/MS measurements. The corresponding dipeptide of the extracted ion electropherograms (EIEs) can be found in Supplementary Table 15 at the end of this section.


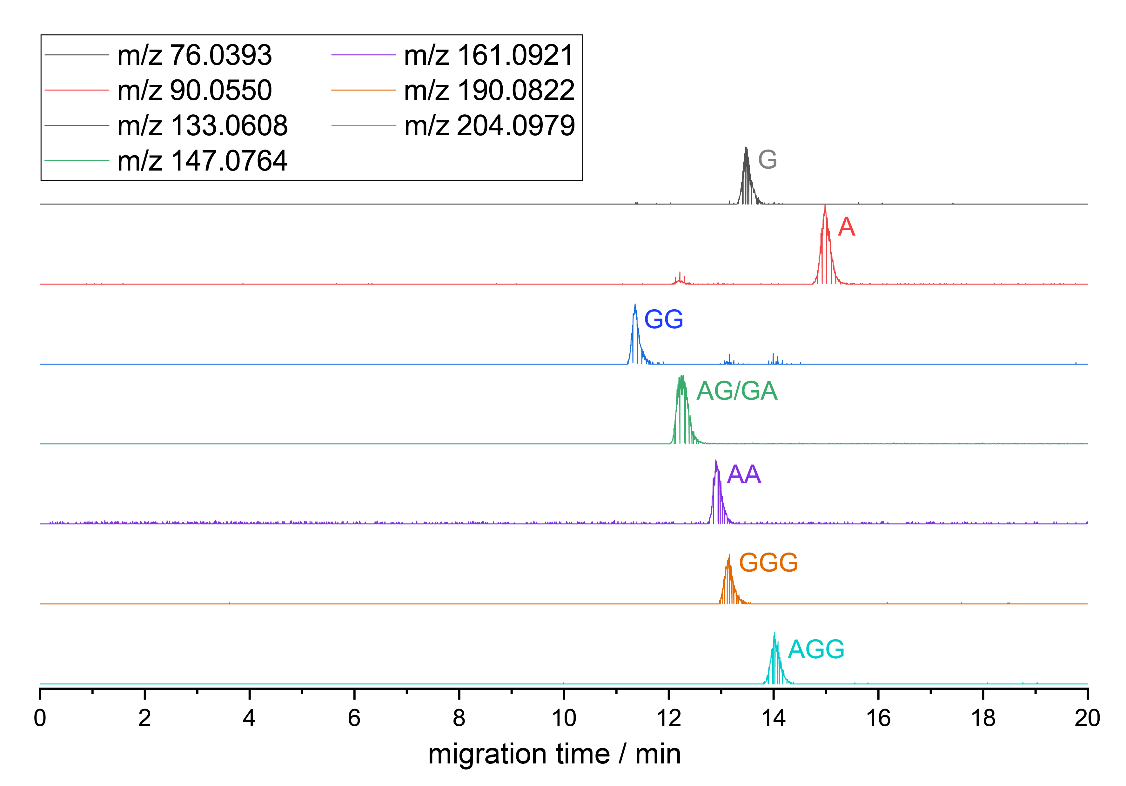


Supplementary Figure 37: EIEs of reference peptides of CE-MS measurements with data dependent MS/MS-experiments. LPA-coated capillary, 70 cm.

Supplementary Figure 38: EIEs of reference diketopiperazines of diglycine and dialanine (both 200 µM).


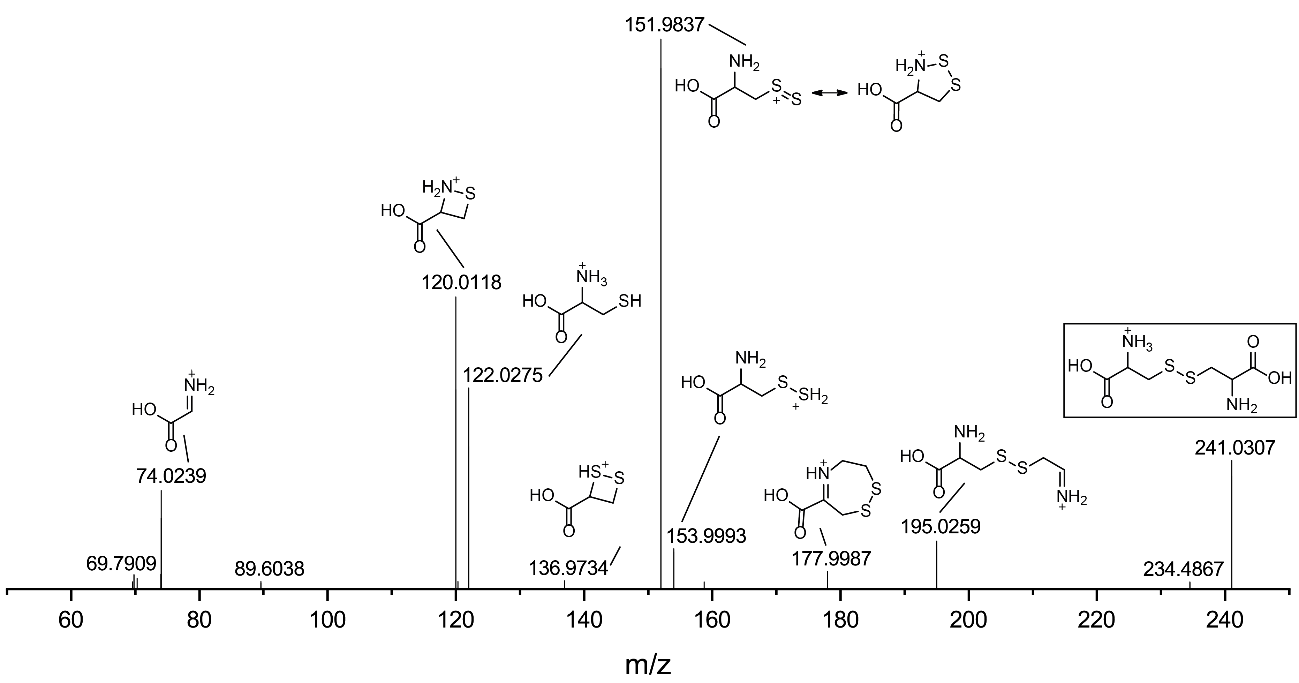


Supplementary Figure 39: Assignment of fragment ions in the MS/MS spectra of cystine after collision induced dissociation (NCE 30 %) of the peak *m/z* = 241.0309 at t_mig_ = 22.2 min after capillary electrophoretic separation.


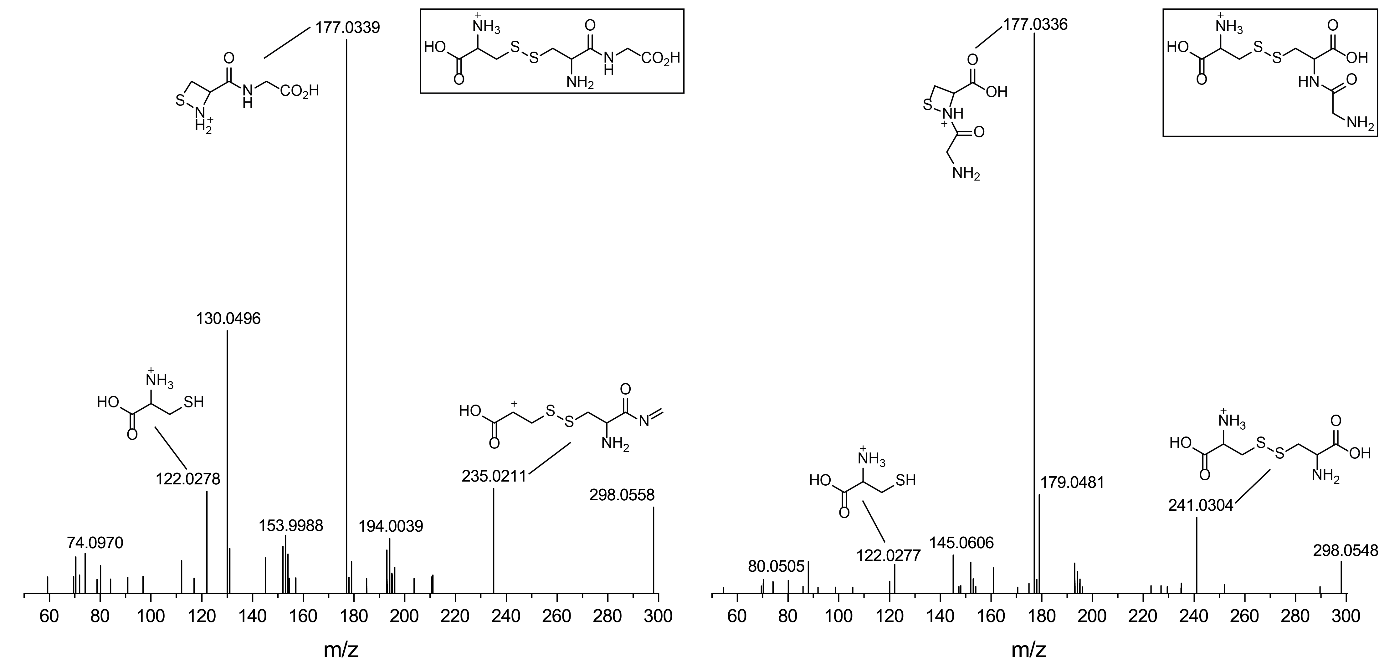


Supplementary Figure 40: Assignment of fragment ions in the MS/MS spectra of cystinylglycine (left) and glycylcystine (right) after collision induced dissociation (NCE 30 %) of the peaks *m/z* = 298.0522 at t_mig_ = 15.7 min and t_mig_ = 16.3 min after capillary electrophoretic separation.

AAA

GAA

GGA

GGG

Supplementary Figure 41: EIEs and peak areas of formed tripeptides after 7 d peptide condensation in SO_2_ with 50 mM initial reactant concentration.

GGA

GAA

GGG

AAA

Supplementary Figure 42: EIEs and peak areas of formed tripeptides after 7 d peptide condensation in SO_2_ with 100 mM initial reactant concentration.

GAA

AAA

GGG

GGA

GAA

GGA

GGG

AAA

Supplementary Figure 43: EIEs and peak areas of formed tripeptides after 7 d peptide condensation in SO_2_ with 200 mM initial reactant concentration.

Supplementary Figure 44: EIEs and peak areas of formed tripeptides after 7 d peptide condensation in SO_2_ with 400 mM initial reactant concentration.

Supplementary Figure 45: Total ion electropherogram of the copper(II)-catalysed SIPF in H_2_O after 7 d starting from the non-polar amino acid mixture. 15 mbar assisting pressure during measurements.

Supplementary Figure 46: EIEs of frequently formed dipeptides after 7 d of SIPF in H_2_O starting from the non-polar amino acid mixture. 15 mbar assisting pressure during measurements.

Supplementary Figure 47: EIEs of frequently formed dipeptides after 7 d of SIPF in H_2_O starting from the non-polar amino acid mixture. 15 mbar assisting pressure during measurements.

Supplementary Figure 48: EIEs of frequently formed dipeptides after 7 d of SIPF in H_2_O starting from the non-polar amino acid mixture. 15 mbar assisting pressure during measurements.

Supplementary Figure 49: Total ion electropherogram of the copper(II)-catalysed peptide condensation in SO_2_ after 21 d starting from the non-polar amino acid mixture.

Supplementary Figure 50: EIEs of frequently formed dipeptides after 21 d peptide condensation in SO_2_ starting from the non-polar amino acid mixture.

Supplementary Figure 51: EIEs of frequently formed dipeptides after 21 d peptide condensation in SO_2_ starting from the non-polar amino acid mixture.

Supplementary Figure 52: EIEs of frequently formed dipeptides after 21 d peptide condensation in SO_2_ starting from the non-polar amino acid mixture.

Supplementary Figure 53: Total ion electropherogram of the copper(II)-catalysed SIPF in H_2_O after 7 d starting from the polar, neutral amino acid mixture. 15 mbar assisting pressure during measurements.

Supplementary Figure 54: EIEs of frequently formed dipeptides after 7 d of SIPF in H_2_O starting from the polar, neutral amino acid mixture. 15 mbar assisting pressure during measurements.

Supplementary Figure 55: EIEs of frequently formed dipeptides after 7 d of SIPF in H_2_O starting from the polar, neutral amino acid mixture. 15 mbar assisting pressure during measurements.

Supplementary Figure 56: EIEs of frequently formed dipeptides after 7 d of SIPF in H_2_O starting from the polar, neutral amino acid mixture. 15 mbar assisting pressure during measurements.

Supplementary Figure 57: Total ion electropherogram of the copper(II)-catalysed peptide condensation in SO_2_ after 21 d starting from the polar, neutral amino acid mixture.

Supplementary Figure 58: EIEs of frequently formed dipeptides after 21 d peptide condensation in SO_2_ starting from the polar, neutral amino acid mixture.

Supplementary Figure 59: EIEs of frequently formed dipeptides after 21 d peptide condensation in SO_2_ starting from the polar, neutral amino acid mixture.

Supplementary Figure 60: EIEs of frequently formed dipeptides after 21 d peptide condensation in SO_2_ starting from the polar, neutral amino acid mixture.

Supplementary Figure 61: Total ion electropherogram of the copper(II)-catalysed SIPF in H_2_O after 7 d starting from the basic amino acid mixture. 15 mbar assisting pressure during measurements.

Supplementary Figure 62: EIEs of frequently formed dipeptides after 7 d of SIPF in H_2_O starting from the basic amino acid mixture. 15 mbar assisting pressure during measurements.

Supplementary Figure 63: Total ion electropherogram of the copper(II)-catalysed peptide condensation in SO_2_ after 21 d starting from the basic amino acid mixture.

Supplementary Figure 64: EIEs of frequently formed dipeptides after 21 d peptide condensation in SO_2_ starting from the basic amino acid mixture.

Supplementary Figure 65: Total ion electropherogram of the copper(II)-catalysed SIPF in H_2_O after 7 d starting from the acidic amino acid mixture. 20 mbar assisting pressure during measurements.

Supplementary Figure 66: EIEs of frequently formed dipeptides after 7 d of SIPF in H_2_O starting from the acidic amino acid mixture. 20 mbar assisting pressure during measurements.

Supplementary Figure 67: Total ion electropherogram of the copper(II)-catalysed peptide condensation in SO_2_ after 21 d starting from the acidic amino acid mixture.

Supplementary Figure 68: EIEs of frequently formed dipeptides after 21 d peptide condensation in SO_2_ starting from the acidic amino acid mixture.

Supplementary Figure 69: Total ion electropherogram of the copper(II)-catalysed SIPF in H_2_O after 7 d starting from the prebiotic amino acid mixture. No assisting pressure during measurements.

Supplementary Figure 70: EIEs of frequently formed dipeptides after 7 d of SIPF in H_2_O starting from the prebiotic amino acid mixture. No assisting pressure during measurements.

Supplementary Figure 71: EIEs of frequently formed dipeptides after 7 d of SIPF in H_2_O starting from the prebiotic amino acid mixture. No assisting pressure during measurements.

Supplementary Figure 72: EIEs of frequently formed dipeptides after 7 d of SIPF in H_2_O starting from the prebiotic amino acid mixture. No assisting pressure during measurements.

Supplementary Figure 73: EIEs of frequently formed dipeptides after 7 d of SIPF in H_2_O starting from the prebiotic amino acid mixture. No assisting pressure during measurements.

Supplementary Figure 74: Total ion electropherogram of the copper(II)-catalysed peptide condensation in SO_2_ after 21 d starting from the prebiotic amino acid mixture.

Supplementary Figure 75: EIEs of frequently formed dipeptides after 21 d peptide condensation in SO_2_ starting from the prebiotic amino acid mixture.

Supplementary Figure 76: EIEs of frequently formed dipeptides after 21 d peptide condensation in SO_2_ starting from the prebiotic amino acid mixture.

Supplementary Figure 77: EIEs of frequently formed dipeptides after 21 d peptide condensation in SO_2_ starting from the prebiotic amino acid mixture.

Supplementary Figure 78: EIEs of frequently formed dipeptides after 21 d peptide condensation in SO_2_ starting from the prebiotic amino acid mixture.

Supplementary Figure 79: Total ion electropherogram of the copper(II)-catalysed SIPF in H_2_O after 7 d starting from the total amino acid mixture. No assisting pressure during measurements.

Supplementary Figure 80: EIEs of frequently formed dipeptides after 7 d of SIPF in H_2_O starting from the total amino acid mixture. No assisting pressure during measurements.

Supplementary Figure 81: EIEs of frequently formed dipeptides after 7 d of SIPF in H_2_O starting from the total amino acid mixture. No assisting pressure during measurements.

Supplementary Figure 82: EIEs of frequently formed dipeptides after 7 d of SIPF in H_2_O starting from the total amino acid mixture. No assisting pressure during measurements.

Supplementary Figure 83: EIEs of frequently formed dipeptides after 7 d of SIPF in H_2_O starting from the total amino acid mixture. No assisting pressure during measurements.

Supplementary Figure 84: EIEs of frequently formed dipeptides after 7 d of SIPF in H_2_O starting from the total amino acid mixture. No assisting pressure during measurements.

Supplementary Figure 85: EIEs of frequently formed dipeptides after 7 d of SIPF in H_2_O starting from the total amino acid mixture. No assisting pressure during measurements.

Supplementary Figure 86: EIEs of frequently formed dipeptides after 7 d of SIPF in H_2_O starting from the total amino acid mixture. No assisting pressure during measurements.

Supplementary Figure 87: EIEs of frequently formed dipeptides after 7 d of SIPF in H_2_O starting from the total amino acid mixture. No assisting pressure during measurements.

Supplementary Figure 88: EIEs of frequently formed dipeptides after 7 d of SIPF in H_2_O starting from the total amino acid mixture. No assisting pressure during measurements.

Supplementary Figure 89: EIEs of frequently formed dipeptides after 7 d of SIPF in H_2_O starting from the total amino acid mixture. No assisting pressure during measurements.

Supplementary Figure 90: EIEs of frequently formed dipeptides after 7 d of SIPF in H_2_O starting from the total amino acid mixture. No assisting pressure during measurements.

Supplementary Figure 91: Total ion electropherogram of the copper(II)-catalysed peptide condensation in SO_2_ after 21 d starting from the total amino acid mixture.

Supplementary Figure 92: EIEs of frequently formed dipeptides after 21 d peptide condensation in SO_2_ starting from the total amino acid mixture.

Supplementary Figure 93: EIEs of frequently formed dipeptides after 21 d peptide condensation in SO_2_ starting from the total amino acid mixture.

Supplementary Figure 94: EIEs of frequently formed dipeptides after 21 d peptide condensation in SO_2_ starting from the total amino acid mixture.

Supplementary Figure 95: EIEs of frequently formed dipeptides after 21 d peptide condensation in SO_2_ starting from the total amino acid mixture.

Supplementary Figure 96: EIEs of frequently formed dipeptides after 21 d peptide condensation in SO_2_ starting from the total amino acid mixture.

Supplementary Figure 97: EIEs of frequently formed dipeptides after 21 d peptide condensation in SO_2_ starting from the total amino acid mixture.

Supplementary Figure 98: EIEs of frequently formed dipeptides after 21 d peptide condensation in SO_2_ starting from the total amino acid mixture.

Supplementary Figure 99: EIEs of frequently formed dipeptides after 21 d peptide condensation in SO_2_ starting from the total amino acid mixture.

Supplementary Figure 100: EIEs of frequently formed dipeptides after 21 d peptide condensation in SO_2_ starting from the total amino acid mixture.

Supplementary Figure 101: EIEs of frequently formed dipeptides after 21 d peptide condensation in SO_2_ starting from the total amino acid mixture.

Supplementary Figure 102: EIEs of frequently formed dipeptides after 21 d peptide condensation in SO_2_ starting from the total amino acid mixture.

Supplementary Figure 103: Total ion electropherogram of the covellite-catalysed peptide condensation in SO_2_ after 7 d.

Supplementary Figure 104: EIEs of formed dipeptides after 7 d peptide condensation in SO_2_ using covellite as catalyst.

| *Dipeptide* | *Exact mass / Da* |  | *Dipeptide* | *Exact mass / Da* |  | *Dipeptide* | *Exact mass / Da* |
| --- | --- | --- | --- | --- | --- | --- | --- |
| **G** | 76.0393 |  | **HG** | 213.0982 |  | **LI** | 245.1860 |
| **A** | 90.0550 |  | **PP** | 213.1234 |  | **LL** | 245.1860 |
| **S** | 106.0499 |  | **VP** | 215.1390 |  | **NI** | 246.1448 |
| **P** | 116.0706 |  | **TP** | 217.1183 |  | **NL** | 246.1448 |
| **V** | 118.0863 |  | **VV** | 217.1547 |  | **VQ** | 246.1448 |
| **T** | 120.0655 |  | **QA** | 218.1135 |  | **RA** | 246.1561 |
| **C** | 122.0270 |  | **KA** | 218.1499 |  | **VK** | 246.1812 |
| **I/L** | 132.1019 |  | **PC** | 219.0798 |  | **NN** | 247.1037 |
| **N** | 133.0608 |  | **EA** | 219.0976 |  | **PM** | 247.1111 |
| **GG** | 133.0608 |  | **SI** | 219.1339 |  | **ID** | 247.1289 |
| **D** | 134.0448 |  | **SL** | 219.1339 |  | **LD** | 247.1289 |
| **Q** | 147.0764 |  | **VT** | 219.1339 |  | **VE** | 247.1289 |
| **GA** | 147.0764 |  | **SN** | 220.0928 |  | **ND** | 248.0877 |
| **K** | 147.1128 |  | **SD** | 221.0768 |  | **TQ** | 248.1241 |
| **E** | 148.0604 |  | **MA** | 221.0954 |  | **TK** | 248.1605 |
| **M** | 150.0583 |  | **VC** | 221.0954 |  | **DD** | 249.0717 |
| **H** | 156.0768 |  | **TT** | 221.1132 |  | **TE** | 249.1081 |
| **AA** | 161.0921 |  | **TC** | 223.0747 |  | **VM** | 249.1267 |
| **SG** | 163.0713 |  | **GF** | 223.1077 |  | **QC** | 250.0856 |
| **F** | 166.0863 |  | **CC** | 225.0362 |  | **KC** | 250.1220 |
| **PG** | 173.0921 |  | **HA** | 227.1139 |  | **EC** | 251.0696 |
| **VG** | 175.1077 |  | **PI** | 229.1547 |  | **TM** | 251.1060 |
| **R** | 175.1190 |  | **PL** | 229.1547 |  | **MC** | 253.0675 |
| **SA** | 177.0870 |  | **PN** | 230.1135 |  | **YA** | 253.1183 |
| **TG** | 177.0870 |  | **PD** | 231.0976 |  | **SF** | 253.1183 |
| **GC** | 179.0485 |  | **VI** | 231.1703 |  | **PH** | 253.1295 |
| **Y** | 182.0812 |  | **VL** | 231.1703 |  | **VH** | 255.1452 |
| **PA** | 187.1077 |  | **VN** | 232.1292 |  | **TH** | 257.1244 |
| **VA** | 189.1234 |  | **RG** | 232.1404 |  | **HC** | 259.0860 |
| **IG** | 189.1234 |  | **VD** | 233.1132 |  | **QI** | 260.1605 |
| **LG** | 189.1234 |  | **TI** | 233.1496 |  | **QL** | 260.1605 |
| **NG** | 190.0822 |  | **TL** | 233.1496 |  | **KI** | 260.1969 |
| **GD** | 191.0663 |  | **TN** | 234.1085 |  | **LK** | 260.1969 |
| **TA** | 191.1026 |  | **SQ** | 234.1085 |  | **QN** | 261.1193 |
| **CA** | 193.0642 |  | **SK** | 234.1448 |  | **IE** | 261.1445 |
| **SS** | 193.0819 |  | **TD** | 235.0925 |  | **LE** | 261.1445 |
| **SP** | 203.1026 |  | **SE** | 235.0925 |  | **NK** | 261.1557 |
| **IA** | 203.1390 |  | **IC** | 235.1111 |  | **QD** | 262.1034 |
| **LA** | 203.1390 |  | **LC** | 235.1111 |  | **NE** | 262.1034 |
| **NA** | 204.0979 |  | **NC** | 236.0700 |  | **WG** | 262.1186 |
| **QG** | 204.0979 |  | **DC** | 237.0540 |  | **KD** | 262.1398 |
| **KG** | 204.1343 |  | **SM** | 237.0904 |  | **SR** | 262.1510 |
| **DA** | 205.0819 |  | **FA** | 237.1234 |  | **ED** | 263.0874 |
| **GE** | 205.0819 |  | **YG** | 239.1026 |  | **PF** | 263.1390 |
| **W** | 205.0972 |  | **SH** | 243.1088 |  | **MI** | 263.1424 |
| **VS** | 205.1183 |  | **QP** | 244.1292 |  | **ML** | 263.1424 |
| **MG** | 207.0798 |  | **PK** | 244.1656 |  | **NM** | 264.1013 |
| **TS** | 207.0976 |  | **PE** | 245.1132 |  | **MD** | 265.0853 |
| **SC** | 209.0591 |  | **II** | 245.1860 |  | **VF** | 265.1547 |

Supplementary Table 15: Complete list of the exact masses of each proteinogenic amino acid and every possible dipeptide combination.

| *Dipeptide* | *Exact mass* |  | *Dipeptide* | *Exact mass* |  | *Dipeptide* | *Exact mass* |
| --- | --- | --- | --- | --- | --- | --- | --- |
| **TF** | 267.1339 |  | **YT** | 283.1288 |  | **WC** | 308.1064 |
| **FC** | 269.0955 |  | **QH** | 284.1353 |  | **YQ** | 310.1397 |
| **YS** | 269.1132 |  | **KH** | 284.1717 |  | **YK** | 310.1761 |
| **IH** | 269.1608 |  | **YC** | 285.0904 |  | **YE** | 311.1238 |
| **LH** | 269.1608 |  | **HE** | 285.1194 |  | **RH** | 312.1779 |
| **NH** | 270.1197 |  | **MH** | 287.1173 |  | **YM** | 313.1217 |
| **HD** | 271.1037 |  | **RI** | 288.2030 |  | **FF** | 313.1547 |
| **RP** | 272.1717 |  | **RL** | 288.2030 |  | **WI** | 318.1812 |
| **VR** | 274.1874 |  | **RN** | 289.1619 |  | **WL** | 318.1812 |
| **QQ** | 275.1350 |  | **RD** | 290.1459 |  | **YH** | 319.1401 |
| **QK** | 275.1714 |  | **WS** | 292.1292 |  | **WN** | 319.1401 |
| **KK** | 275.2078 |  | **HH** | 293.1357 |  | **WD** | 320.1241 |
| **QE** | 276.1190 |  | **QF** | 294.1448 |  | **RF** | 322.1874 |
| **WA** | 276.1343 |  | **KF** | 294.1812 |  | **YF** | 329.1496 |
| **KE** | 276.1554 |  | **FE** | 295.1289 |  | **RR** | 331.2201 |
| **TR** | 276.1666 |  | **YI** | 295.1652 |  | **WQ** | 333.1557 |
| **EE** | 277.1030 |  | **YL** | 295.1652 |  | **WK** | 333.1921 |
| **QM** | 278.1169 |  | **YN** | 296.1241 |  | **WE** | 334.1398 |
| **RC** | 278.1282 |  | **YD** | 297.1081 |  | **WM** | 336.1377 |
| **MK** | 278.1533 |  | **MF** | 297.1268 |  | **YR** | 338.1823 |
| **ME** | 279.1009 |  | **WP** | 302.1499 |  | **WH** | 342.1561 |
| **YP** | 279.1339 |  | **HF** | 303.1452 |  | **YY** | 345.1445 |
| **IF** | 279.1703 |  | **RQ** | 303.1775 |  | **WF** | 352.1656 |
| **LF** | 279.1703 |  | **RK** | 303.2139 |  | **WR** | 361.1983 |
| **NF** | 280.1292 |  | **RE** | 304.1616 |  | **YW** | 368.1605 |
| **MM** | 281.0988 |  | **WV** | 304.1656 |  | **WW** | 391.1765 |
| **FD** | 281.1132 |  | **WT** | 306.1448 |  |  |  |
| **YV** | 281.1496 |  | **RM** | 306.1594 |  |  |  |

Supplementary Table 15: Complete list of the exact masses of each proteinogenic amino acid and every possible dipeptide combination.

# Extracted ion chromatograms and mass spectra


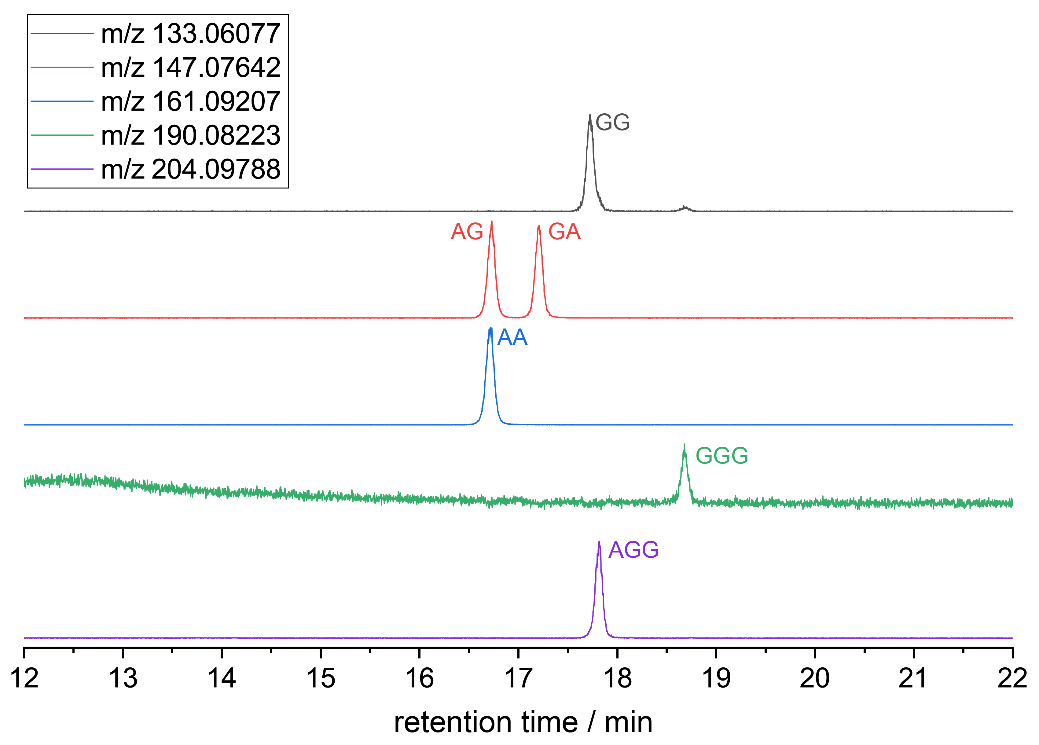


Supplementary Figure 105: Extracted ion chromatograms of reference peptides after HPLC separation using a Daicel DCpak PTZ column (4.6x150 mm, 3 µm), flow 0.9 mL/min, T = 30 °C, A: 20 mmol/L NH_4_OAc_(aq)_, B: ACN, gradient: 0-10 min 70 % B, 10-25 min 70-0 % B, 25-35 min 0 % B.


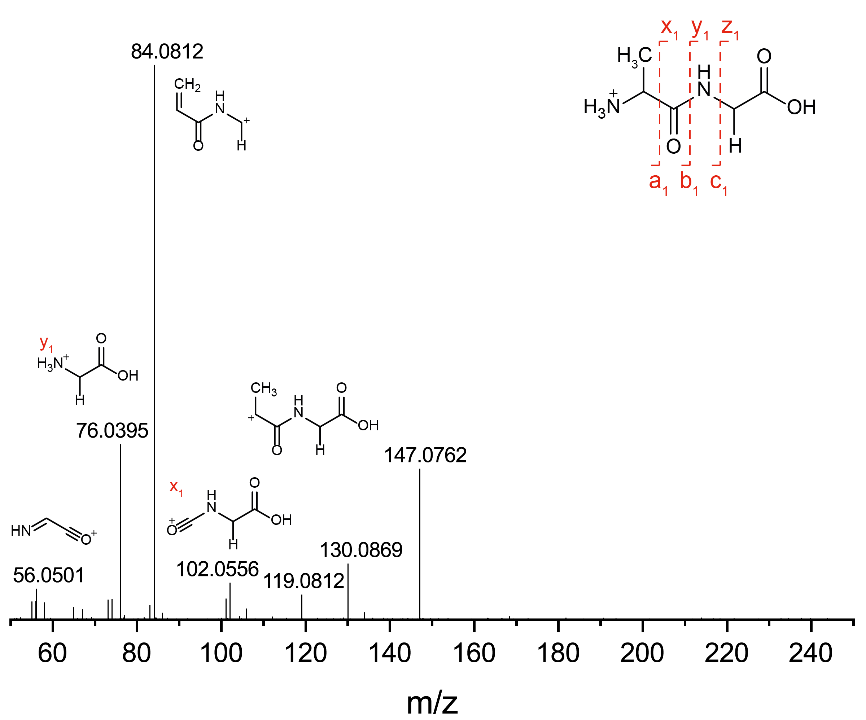


Supplementary Figure 106: MS/MS spectrum of alanylglycine after collision induced dissociation (15 eV) of the signal at t_ret_ = 16.7 min of HPLC separation on a Daicel DCpak PTZ solumn (4.6x150 mm, 3 µm), flow 0.9 mL/min, T = 30 °C, A: 20 mmol/L NH_4_OAc_(aq)_, B: ACN, gradient: 0-10 min 70 % B, 10-25 min 70-0 % B, 25-35 min 0 % B.


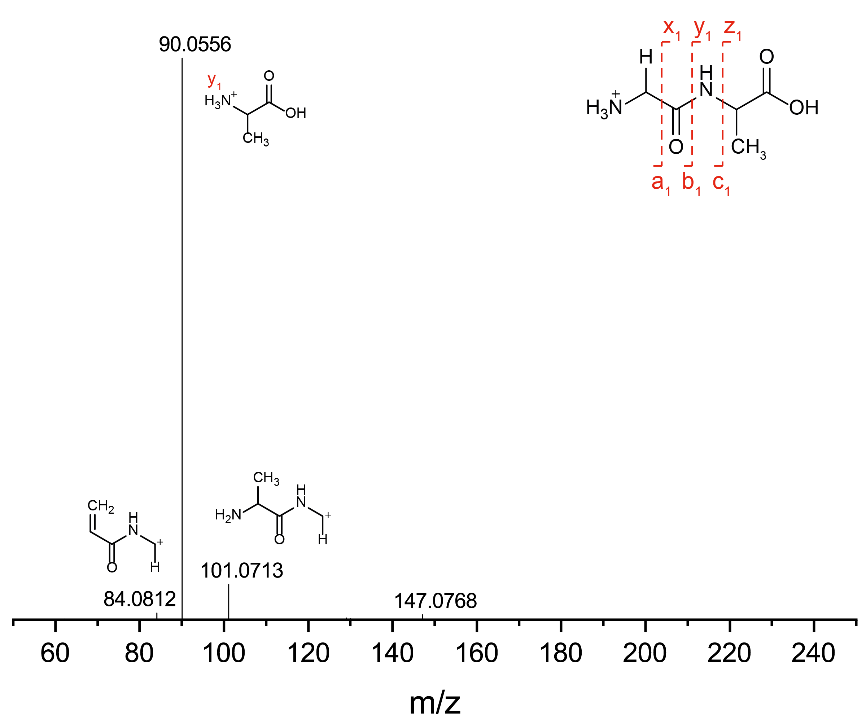


Supplementary Figure 107: MS/MS spectrum of glycylalanine after collision induced dissociation (15 eV) of the signal at t_ret_ = 17.1 min of HPLC separation on a Daicel DCpak PTZ solumn (4.6x150 mm, 3 µm), flow 0.9 mL/min, T = 30 °C, A: 20 mmol/L NH_4_OAc_(aq)_, B: ACN, gradient: 0-10 min 70 % B, 10-25 min 70-0 % B, 25-35 min 0 % B.

# References

1. . Zhu, G., Sun, L. & Dovichi, N. J. Thermally-initiated free radical polymerization for reproducible production of stable linear polyacrylamide coated capillaries, and their application to proteomic analysis using capillary zone electrophoresis–mass spectrometry. *Talanta* **146,** 839-843 (2016). [↑](#endnote-ref-1)
